# Optimal CD8^+^ T cell effector function requires costimulation-induced RNA-binding proteins that reprogram the transcript isoform landscape

**DOI:** 10.1038/s41467-022-31228-0

**Published:** 2022-06-20

**Authors:** Timofey A. Karginov, Antoine Ménoret, Anthony T. Vella

**Affiliations:** Department of Immunology, School of Medicine, University of Connecticut, UConn Health, 263 Farmington Avenue, Farmington, CT 06030 USA

**Keywords:** Lymphocyte activation, Molecular biology, T cells, Cellular immunity

## Abstract

Boosting T cell activation through costimulation directs defense against cancer and viral infections. Despite multiple studies targeting costimulation in clinical trials, the increased potency and reprogramming of T cells endowed by costimulation is poorly understood. Canonical dogma states that transcription mediates T cell activation. Here, we show that the spliceosome, controlling post-transcriptional alternative splicing and alternative polyadenylation, is the most enriched pathway in T cells after CD134/CD137 costimulation. Costimulation of CD8+ T cells significantly increases expression of 29 RNA-binding proteins while RNA-seq uncovers over 1000 differential alternative splicing and polyadenylation events. Using in vivo mouse and in vitro human models, we demonstrate that RNA-binding protein Tardbp is required for effector cytokine production, CD8+ T cell clonal expansion, and isoform regulation after costimulation. The prospect of immune response optimization through reprogramming of mRNA isoform production offered herein opens new avenues for experimentally and therapeutically tuning the activities of T cells.

## Abstract (web summary)

The provision of costimulatory signals to T cells are known to be vital in the direction and induction of a T cell driven response. Here the authors show an optimal T cell response requires a costimulatory induced modification of the transcript landscape and achieves this via modulation of the RNA binding proteins.

## Introduction

Costimulation of CD8^+^ T cells induces their differentiation into effector cells contributing to inflammatory physiological and pathological response^1^. Two costimulatory signals, TNF superfamily receptors CD134 (OX40) and CD137 (41BB), are expressed on activated CD8^+^ T cells and are triggered in both protective and pathological conditions to induce differentiation into surviving T effector cells^2–6^. Textbook immunology stipulates that T cell costimulation induces upregulation of many transcriptional pathways including JAK-STAT, mTOR, and NF-kB that contribute to inflammatory cytokine production (IFNγ, TNF, and IL-2) and clonal expansion^1^.

Although costimulation increases the level of some transcripts for T cell activation, decades of work have indicated significantly greater complexity to RNA regulation in T cell activation. Namely, that changes in RNA splicing and polyadenylation take place during T cell differentiation and activation^7–10^. RNA splicing has been studied in development, neurons, viruses, and cancers, among other systems, for over 40 years^11,12^. During this time, a critical distinction was made between constitutive RNA splicing —referring to the removal of introns to produce a mature mRNA product ready for translation—versus alternative RNA splicing—referring to the removal of introns and specific exon regions that alter the final mRNA product to an isoform of the original gene^13^. Further classifications divided types of alternative splicing (AS) to exon skipping, intron retention, and alternative 3′/5′ end splicing^14^. New insights revealed modifications to the polyadenylation sites found in the 3′UTR and in intronic regions that change the length of the pre-mRNA product—termed alternative polyadenylation (APA)^15^. AS and APA have since been shown to regulate transcript abundance, mRNA stability, mRNA export, and translation efficiency in cells^16^.

Despite the emerging knowledge in RNA splicing regulation, few studies have mechanistically explored RNA splicing in immune cells and even fewer have shown the role of RNA binding proteins regulating T effector function in vivo. Studies that have explored these points suggest several principles. First, changes in immune cell state lead to different alternative splicing patterns^17–19^. Second, intronic and 3′UTR alternative polyadenylation play a role in diversifying immune cell transcriptomes^10,20^. Third, key immune genes are modified by the process of alternative RNA splicing, changing transcript expression, and producing isoforms with differing functions. Fourth, specific RBPs can regulate the immune response^21^. Whether RNA splicing is necessary and/or sufficient for immune cell function and differentiation, which genes are spliced, which splicing patterns regulate immune cell states, which immune signals alter the transcript isoform landscape, and which RNA binding proteins are key players for immune cells in physiology and pathology still remain unclear.

Here we show that the spliceosome is the most significantly enriched pathway in CD8^+^ T cells receiving costimulation. Further, we show that costimulation induces significant AS and APA changes in known T effector genes. By using an adapted CRISPR/Cas9 model paired with in vivo adoptive transfer of CD8^+^ T cells, we demonstrate that RNA binding protein (RBP) *Tardbp*, which controls AS and APA, is necessary for optimal CD8^+^ T effector function after costimulation^22^. These studies demonstrate the necessity of RBPs controlling AS and APA for full CD8^+^ T effector activity. Thus, this study pinpoints a role for RNA splicing as an RNA regulatory mechanism essential for in vivo CD8^+^ T cell effector function.

## Results

### Costimulation of CD8^+^ T cells upregulates a unique signature of RBPs critical for AS and APA

**Fig. 1:**
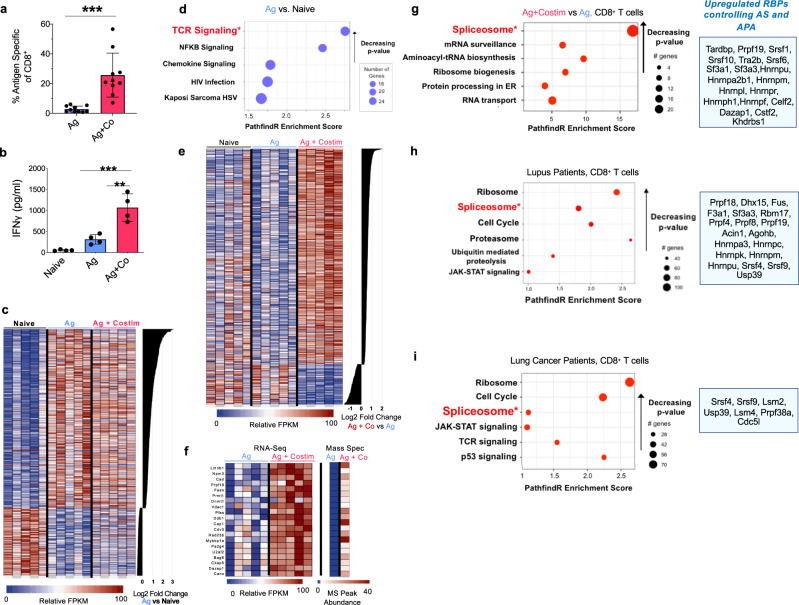
Costimulation of CD8^+^ T cells upregulates a unique genetic signature of RNA binding proteins critical for alternative RNA splicing and alternative polyadenylation. **a** Percent OT-I, CD45.1^+^ cells of CD8^+^ T cell from spleen and lymph nodes 3.5 days after receiving antigen alone or antigen with costimulation as described in Supplementary Fig. 1. Data were pooled from two independent experiments. Each data point represents a biological replicate, *n* = 10. **b** ELISA for IFNγ secretion after restimulation of sorted, purified OT-I cells with PMAi for 20 h *n* = 4/group. Data is representative of 1 of 2 independent experiments. **c**. Relative FPKM levels of the top700 genes ranked by log_2_fold change between antigen and naïve groups (FDR < 0.05), *n* = 5/group. Data is representative of 1 of 2 independent experiments where each column represents an individual biological replicate. **d** Pathway analysis of 700 genes identified in 1c ranked by log_2_fold change between Ag alone and Naïve (blue) (**e**) Relative FPKM levels of 700 genes ranked by log_2_fold change (FDR < 0.05) between Ag+Costim and Ag alone groups, *n* = 5/group. Data is representative of 1 of 2 independent experiments where each column represents an individual biological replicate. **f** Top 20 (of 274) genes found to be significantly upregulated expressed between Ag+Costim and Ag alone groups in both RNA & mass spectrometry experiments (FDR < 0.05), *n* = 5/group. **g** Pathway analysis of 274 genes identified in **d** ranked by log_2_fold change between Ag+Costim and Ag alone groups. The blue panel shows 19 upregulated RBPs regulating AS and APA out of 118 genes and proteins found upregulated across 2 datasets (Supplementary Fig. 1) between Ag+Costim and Ag alone groups, *n* = 10/group across 2 datasets for RNA, *n* = 2 pooled from 10 samples across 2 datasets for mass spectrometry. **h** Pathway analysis of genes identified as significantly differentially expressed (FDR < 0.05) in CD8^+^ T cells from blood of lupus patients compared to healthy controls and 19 RBPs upregulated between these groups, *n* > 14/group. **i** Pathway analysis of genes identified as significantly differentially expressed (FDR < 0.05) in intratumoral PD1^+^ CD8^+^ T cells patients compared to intratumoral PD1^−^ CD8^+^ T cells and 7 RBPs upregulated between these groups, *n* = 11/group. Data are presented as mean values +/− SD using a two-sided t test with Benjimini, Krieger, and Yekutieli to control FDR where * represents n digits after decimal for *p* value. Source data are provided as a Source Data file.

To understand how costimulation rewires the molecular pathways of CD8^+^ T cells and contributes to optimal T effector function, we used an in vivo mouse model of adoptively transferred CD8^+^ OT-I T cells receiving antigen (Ag) or antigen with costimulation (Ag+Costim) (Supplementary Fig. 1a). Clonal expansion of antigen-specific CD8^+^ T cells and IFNγ secretion after in vitro restimulation were significantly greater after Ag+Costim compared to Ag alone, indicating that costimulation enhances the effector potential of CD8^+^ T cells and their survival (Fig. 1a, b). RNA-seq analysis of the same CD8^+^ T cells from Fig. 1a and Naïve CD8^+^ T cells comparing the top genes ranked by log_2_ fold change between Ag vs Naive groups (FDR < 0.05) showed RNA expression changes in canonical immune signaling genes after antigen stimulation (Fig. 1c). TCR Signaling, NF-kB Signaling, and Chemokine Signaling were among the top differential pathways between Ag and Naive groups after pathway analysis (Fig. 1d). In our data, key genes in these pathways including NFAT, NF-kB, AP-1, and IL-2 transcripts showed only minor changes (transcripts had less than .5 log_2_fold change) when comparing primed and costimulated cells (Ag+Costim vs. Ag) (Supplementary Fig. 2a). Rather, these transcripts were significantly increased only when comparing antigen primed and naïve cells (Supplementary Fig. 2b). In other words, costimulation only induced a modest change in transcription of NF-kB, AP-1, NFAT, or IL-2. This same pattern was observed in two independent sequencing experiments (Supplementary Figs. 2 and 3).

In order to identify which transcripts were specifically induced by costimulation of CD8^+^ T cells, we filtered for changes between Ag and Ag+Costim groups which demonstrated a unique gene signature distinct from that after priming with antigen alone (Fig. 1e and Supplementary Fig. 3c). To validate these findings, lysates from the same CD8^+^ T cells were analyzed using mass spectrometry. Analysis revealed 274 proteins that were upregulated in Ag+Costim versus Ag alone with RNA expression in 274 genes from Fig. 1e matching increased protein levels (Fig. 1f). This number increased to 925 proteins and genes found upregulated between Ag+Costim and Ag groups in our second replicate of this experiment (Supplementary Fig. 3d). Unexpectedly, the spliceosome was the top enriched pathway by *p*-value, including RBPs with a known role in RNA splicing upregulated in both RNA and protein (Fig. 1g and Supplementary Fig. 3e). The spliceosome includes both constitutive splicing factors responsible for intron removal for mature mRNA formation as well RBPs that carry out alternative splicing and alternative polyadenylation to change mRNA isoform structure and expression. To narrow down reproducibly upregulated RBPs after costimulation, we filtered for genes and proteins that were upregulated in both replicates of our experiment between Ag+Costim and Ag groups. This included 118 genes and 29 RBPs, 19 of which had known alternative RNA splicing or alternative RNA polyadenylation activity based on a literature search (Fig. 1g blue box, Supplementary Data 1)^23–26^. Transcripts of these 19 RBPs were upregulated to a significantly greater level than the canonical costimulation-induced genes of NFkB1, AP-1, IL-2, and NFAT (Supplementary Fig. 2a). Importantly, these RBPs were specifically induced by costimulation as they showed little change in transcript expression after priming alone (Supplementary Fig. 2b). To address the pertinence of RBPs regulating alternative splicing and polyadenylation in human disease, we analyzed two patient datasets of CD8^+^ T cells purified and sequenced directly from patients with lupus without restimulation (GSE97264) and lung cancer (GSE99531)^27,28^. Both showed enrichment for spliceosome genes in the top 3 pathways, raising the hypothesis that upregulation of RBPs is clinically relevant for alternative RNA regulation of CD8^+^ T cells in human disease (Fig. 1h, i blue box). Altogether, our data show that costimulation specifically induces upregulation of RBP transcript and protein that may contribute to its optimization of T effector potential. These RBPs potentially regulate alternative splicing and alternative polyadenylation that contribute to physiological and pathological T cell states.

### AS, APA, and alternative TSS are enriched after costimulation of CD8^+^ T cells

**Fig. 2:**
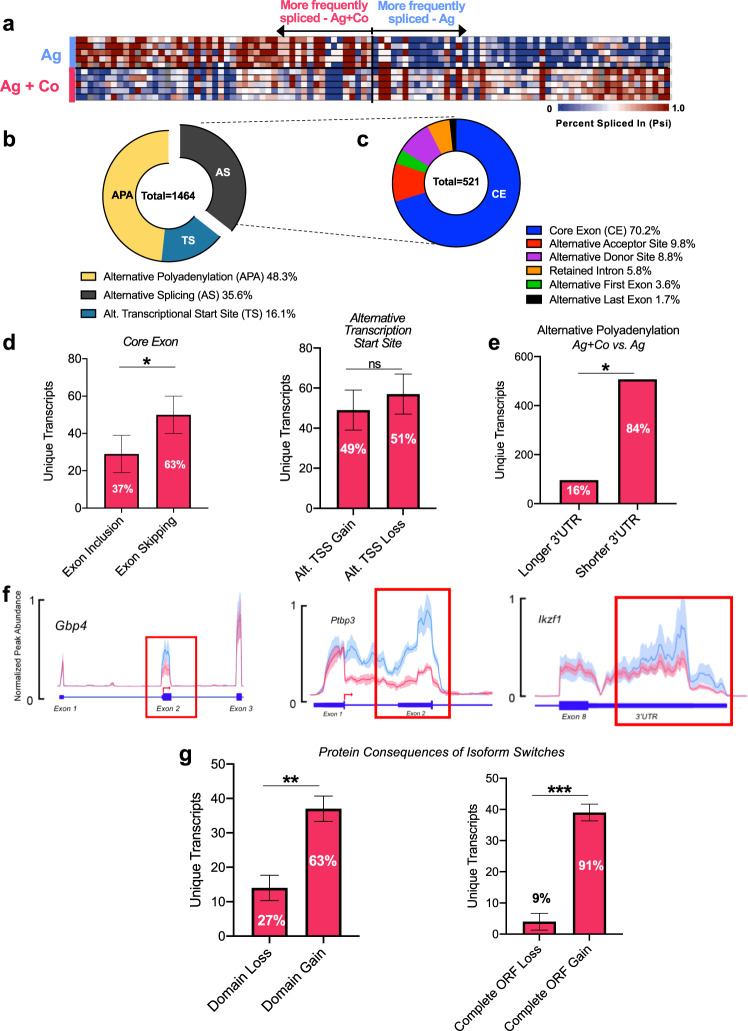
Alternative splicing, alternative polyadenylation, and alternative transcriptional start sites are enriched after costimulation of CD8^+^ T cells. **a** Percent spliced in levels (Psi) of 1464 splice site differences in 950 genes between Ag and Ag+Costim groups (Probability > 0.9, Psi Diff > .05 or <−0.05 after Whippet analysis of deep RNA-seq samples from Fig. 1, comparing antigen+costim and antigen alone groups (100 shown), *n* = 5 samples/group. Data is representative of 1 of 2 independent experiments. **b** Distribution of RNA alterations of events seen in **a**. **c** Distribution of alternative splicing events from (**b**). **d** Isoform switch analysis of splicing sites from deep RNA-seq samples in Fig. 1 showing enrichment of splicing event type between Ag+Costim and Ag groups. Percentages represent a fraction of the total number of exon splicing events between Ag+Costim and Ag groups i.e., 63% of exon splicing events were skipped exons (in the Ag+Co group) while 37% were included exons, *n* = 5 mice/group. **e** Analysis of alternative polyadenylation sites and 3′UTR length between Ag+Costim and Ag groups, *n* = 5 samples/group. **f** Three examples of splicing events from IGV browser tracks of sequencing from (**a**). Peak abundance was normalized to exon nearest the splice site of interest. Shaded area represents standard deviation of *n* = 5 samples/group. Darker line represents the mean for each group. **g** Predicted protein consequences of isoform switches in (**a**), *n* = 5 samples/group. Data are presented as mean values +/− SE derived from two-sided Fisher’s exact test with Benjamini–Hochberg adjustment for multiple comparisons. Source data are provided as a Source Data file.

We hypothesized that upregulation of RBPs after CD8^+^ T cell costimulation should occur with changes in alternative RNA splicing and polyadenylation events. To test this model, we conducted RNA-seq analysis on the same purified CD8^+^ T cells from Naïve, Ag, and Ag+Costim groups as in Fig. 1a–g. Analysis with Whippet identified 1464 changes in transcript isoforms in 950 genes between Ag and Ag+Costim groups (Probability > .9, Percent-spliced in (Psi) difference > 0.05 or <−0.05) (Fig. 2a and Supplementary Data 2)^29^. The distribution of these data uncovered three primary modes of RNA alterations: (1) Alternative polyadenylation (APA) (48.3%) (2) Alternative splicing (AS) (35.6%) and (3) Alternative Transcriptional Start Sites (TS) (16.1%) (Fig. 2b). AS events can be subdivided into specific categories: our analysis showed AS events after costimulation primarily favored core exon splicing (70.2%) but also demonstrated changes in alternative acceptor/donor regions (9.8%/8.8%), intron retention (5.8%), and alternative first/last exon sites (3.6%/1.7%) (Fig. 2c). We then tested if major RNA splice events favored either Ag or Ag+Costim groups by using IsoformSwitchAnalyzeR for core exon splicing and transcriptional start sites and DaPars for alternative polyadenylation^30,31^. The number of unique genes with isoform changes due to core exon splicing significantly favored exon skipping compared to exon inclusion in Ag+Costim group (Fig. 2d, left panel) while alternative transcriptional start site gain and loss was nearly equivalent between groups (Fig. 2d, right panel). Thus, costimulation-induced splicing changes resulted in isoforms that were missing exon regions present during antigen-priming, suggesting these regions could be important in regulating optimal effector cell function. Further, alternative polyadenylation events in the 3′UTR region of genes significantly favored shortening of the 3′UTR in the Ag+Costim group (Fig. 2e). Representative examples of AS, APA, and TS are given in Fig. 2f. Prior work has shown shortened 3′UTRs in activated T cells and resulting changes in RBP interactions^32,33^. Our data further support these observations and suggest an important role for APA in controlling effects of costimulation through 3′UTR regulation. Taken together, these data showed that costimulation of CD8^+^ T cells induces significant changes in AS, APA, and TS to specifically favor exon skipping and 3′UTR shortening.

Further, we hypothesized that splicing changes resulting in differentially expressed transcript isoforms could be used to predict changes to protein domain expression. Thus, we analyzed predicted protein consequences of isoform switches and noted a significant skew towards domain gain and open reading frame (ORF) gain in Ag+Costim groups (Fig. 2g)^30^. We validated the overall pattern of AS and APA changes in a second, independent RNA-seq experiment for costimulated CD8^+^ T cells (Ag+Costim vs Ag) (Supplementary Fig. 4 and Supplementary Data 3). This dataset identified an even greater number of AS and APA events but showed nearly the same skew towards Ag+Costim in specific AS, APA, and protein isoform patterns (Supplementary Fig. 4b–f). Notably, we observed an increase in intron retention and nonsense-mediated decay (NMD) sensitive isoforms in the Ag only group, indicating a predisposition towards non-coding transcripts when costimulation is absent (Supplementary Fig. 4d, f). The observation of increased domain gain was unexpected given the prevalence of exon skipping in costimulated CD8^+^ T cells (Fig. 2d, g and Supplementary Fig. 4d, f). However, this was attributed to a combination of AS events seen in Fig. 2c that led to an increase in coding transcripts with domain and ORF gains as compared with those targeted for NMD (Fig. 2c and Supplementary Fig. 4c, f). In other words, it is possible that without costimulation, exon and intron inclusion leads to non-coding or NMD-sensitive transcripts. Costimulation would lead to an increase of translated transcripts and therefore increase domain gain by removal of these introns and potential poison exon regions that have been previously shown to lead to unproductive transcripts^34^. In total, these data suggest that costimulation induces AS and APA changes to optimize production of transcripts with protein coding potential and thereby enhance the effector function of CD8^+^ T cells.

### RBP *Tardbp* binds to immune-regulatory genes containing splicing events induced by costimulation of CD8^+^ T cells

**Fig. 3:**
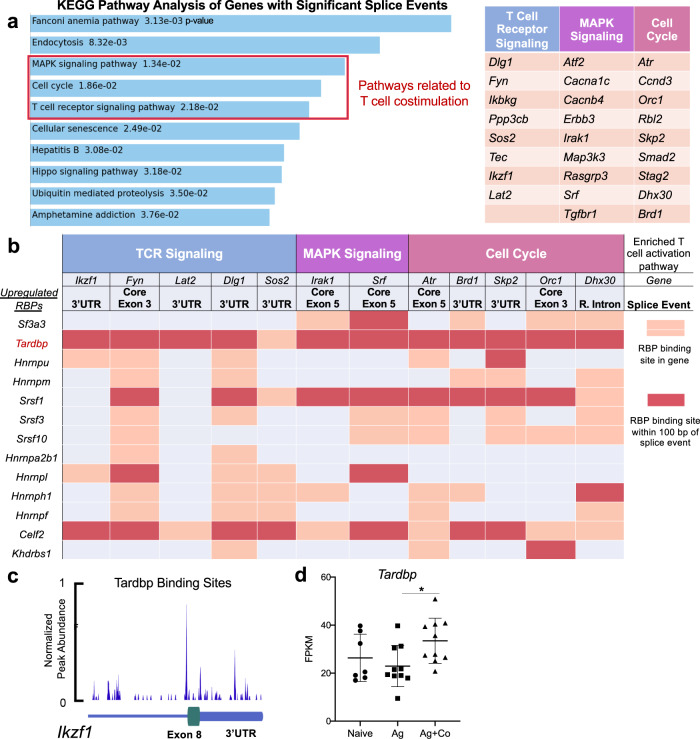
RBP *Tardbp* binds to immune-regulatory genes containing splicing events induced by costimulation of CD8^+^ T cells. **a** KEGG pathway analysis of 950 genes containing 1464 RNA splice events from Ag+Costim vs Ag groups in Fig. 2a ranked by p value as well as 26 genes with splice events grouped by 3 immune-related pathways. **b** Binding of 13 significantly upregulated RBPs from 1g to a representative subset of 12 immune-related splice events found in (**a**). **c** CLIP-seq data from ENCODE database for Tardbp binding to RNA in *Ikzf1*. **d** Relative FPKM of *Tardbp* from two independent RNA-seq datasets comparing naïve, Ag, and Ag+Co CD8^+^ T cells from dataset in Fig. 1 and Supplementary Fig. 2, *n* = 7 for naïve, *n* = 10 for Ag and Ag+Co. Data are presented as mean values +/− SD using a two-sided t test with Benjamini, Krieger and Yekutieli to control FDR where * represents n digits after decimal for *p* value. Source data are provided as a Source Data file.

We next asked which RBPs could be important in regulating the observed AS and APA events after costimulation of CD8^+^ T cells. To answer this, we conducted KEGG pathway analysis of the 1464 RNA splice events in 950 genes shown in Fig. 2a^35^. Enrichment analysis showed three major pathways containing 26 genes specifically related to T cell costimulation, namely MAPK signaling, Cell cycle, and T cell receptor signaling (Fig. 3a). This indicated that costimulation could directly impact AS and APA of downstream pathways known to regulate T cell effector function. We selected all 26 genes with splicing events belonging to these three pathways to identify RBP binding sites (Fig. 3a, right table). We subsequently searched for evidence of binding sites for 13 selected RBPs within the 26 target genes or within 100 bp of the splicing event identified (Fig. 3b). These 13 RBPs were identified by filtering our RNA-seq and MS data of costimulated CD8^+^ T cells (Fig. 1e, f) for RBPs upregulated after costimulation with published evidence of AS or APA regulation. Of 19 upregulated RBPs, 13 had published CLIP-seq data for RNA binding in human cells^36^. *Tardbp* was identified as the only RBP with binding sites in 25 of 26 selected genes with splicing events. Figure 3b, highlights binding sites in 12 of these genes representative of the 26, 11 of which had a *Tardbp* binding site within 100 bp of the splicing event (Fig. 3b). This included specific binding to the 3′UTR and exon of IL-2 signaling and immune-regulatory transcription factor *Ikzf1* (Fig. 3c). Furthermore, *Tardbp* was upregulated after costimulation in RNA-seq data, mass spectrometry, and by immune blot of costimulated CD8^+^ T cells compared to naïve controls (Fig. 3d and Supplementary Fig. 5). In total, our binding analysis, transcriptomics, and protein data highlighted RBP *Tardbp* as an important candidate regulating AS, APA, and TS events observed after costimulation.

### *Tardbp* regulates AS and APA in CD8^+^ T cells

**Fig. 4:**
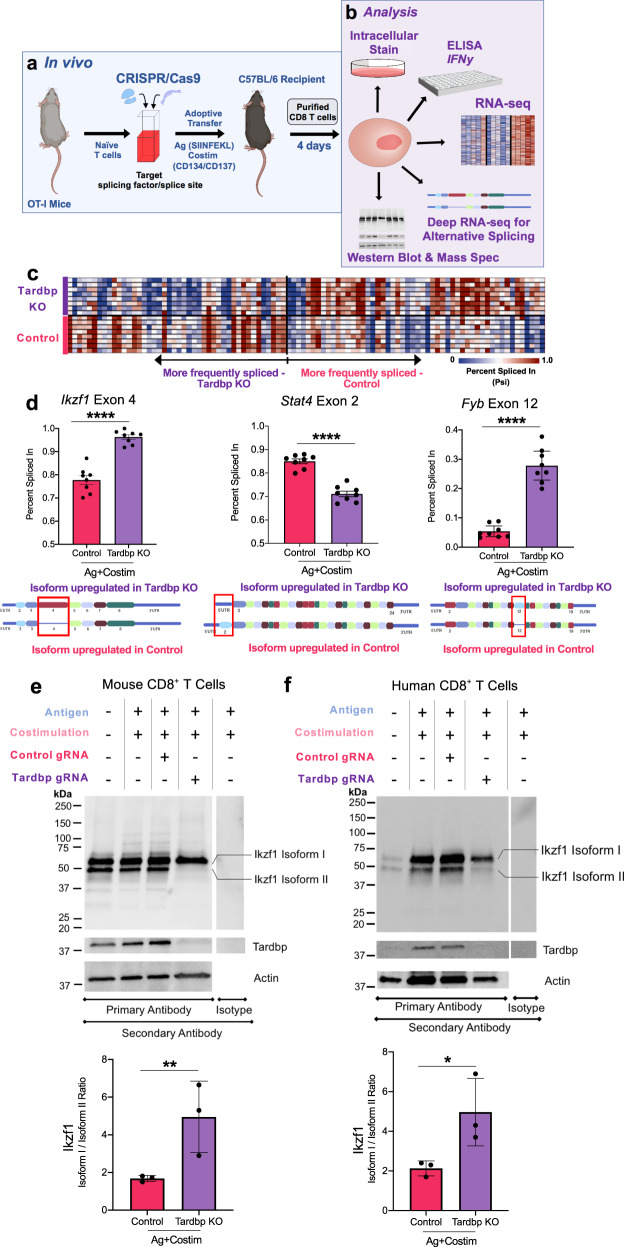
*Tardbp* regulates alternative RNA splicing and alternative polyadenylation in CD8^+^ T cells. **a** Model of in vivo CRISPR/Cas9 knockout and adoptive transfer of CD8^+^ T cells. **b** Analysis of CD8^+^ T cells from model in **a**. **c** Deep RNA-seq of analyzed with Whippet of Control and Tardbp KO CD8^+^ T cells receiving Ag+Co after 3.5 days in vivo. Filtering included probability >0.9, Percent-spliced in (Psi) difference >0.1 or <−0.1; total reads >50; 100 splicing events shown, *n* = 8/group. **d** Examples of specific splicing events from A at Ikzf1 exon 4, Stat4 exon 2, and Fyb exon 12. Representative mRNA isoforms for each splicing event are shown below Psi plots, *n* = 8/group. **e** Western blot for Ikzf1 & Tardbp showing Ctrl & *Tardbp* KO CD8^+^ T cells pooled from (**a**) (*n* = 3 experimental replicates) including quantification of Isoform I to Isoform II ratio below. **f** Western blot for Ikzf1 of human CD8^+^ T cells undergoing CRISPR/Cas9 with Ctrl gRNA and *Tardbp* gRNA 4 days after stimulation CD3/CD28 stimulation (*n* = 3 experimental replicates) including quantification of Isoform I to Isoform II ratio below. Data are presented as mean values +/− SD using a two-sided t test with Benjamini, Krieger, and Yekutieli to control FDR where * represents *n* digits after decimal for *p* value. Source data are provided as a Source Data file.

We identified *Tardbp* as a candidate regulator of CD8^+^ T cell costimulation. However, it was unclear if *Tardbp* mediated RNA splicing changes in activated CD8^+^ T cells. To test this concept, we paired adoptive transfer of CD8^+^ T cells into recipient host mice with a new CRISPR/Cas9 protocol^22^. Specifically, Cas9 and gRNA against *Tardbp* (or control gRNA) were electroporated into naïve OT-I CD8^+^ T cells and then adoptively transferred into recipients followed by immunization with ovalbumin-derived SIINFEKL antigen (Ag) plus costimulation (Fig. 4a). Three and a half days after immunization, these antigen-specific CD8^+^ T cells were purified from 8 individual recipient mice then subjected to stimulation and deep RNA-seq (Fig. 4b). Deep RNA-seq analysis using Whippet uncovered 233 alternatively spliced events in 161 genes (Probability > 0.9, Percent-spliced in (Psi) difference > 0.1 or <−0.1; total reads > 50; 100 events shown) (Fig. 4c and Supplementary Data 4)^29^. Three of the most significant changes in exon skipping included known regulators of TCR signaling after activation, transcription factor *Ikzf1*, transcription factor *Stat4*, and T cell activation adaptor *Fyb* (Fig. 4d). More specifically, *Ikzf1* Exon 4 was included in *Tardbp KO* with a Psi level difference of 18%; this exon splicing event forms Isoform II of *Ikzf1* which is important for the regulation of IL-2 signaling^37^. *Stat4* Exon 2 was differentially spliced out in *Tardbp* KO cells with a Psi difference of 14%; this region codes for a portion of the *Stat4* N-terminal domain, known to be essential for phosphorylation and CD4^+^ T cell activation^38^. Finally, Exon 12 of *Fyb* was included in *Tardbp* KO cells with a Psi difference of 22%; splicing of Exon 12 produces the isoform Fyb-120 which regulates TCR signaling through the NFAT/AP-1 pathway (Fig. 4d)^39^. This region contains multiple regulatory phosphorylation sites for serine/threonine kinases (cAMP/cGMP kinase, protein kinase C)^39^.

Further, we tested whether the splicing activity of *Tardbp* had effects on protein isoform expression. Isoform II of Ikzf1 was nearly absent on immunoblotting in the *Tardbp* KO group, suggesting that splicing by *Tardbp* of Exon 4 of *Ikzf1* resulted in a consequential protein change (Fig. 4e). Probing for Tardbp also validated that this protein was not produced in our CD8^+^ T cells receiving *Tardbp* gRNA (4e). Notably, the pattern of Ikzf1 splicing was different in the absence of costimulation (lane 1 vs lane 4 of Fig. 4e) and levels of Tardbp were higher than in the *Tardbp* KO group. This indicated that while Tardbp was required for splicing of Exon 4 to produce *Ikzf1* isoform II in costimulated cells, a basal level of Tardbp still allows for production of *Ikzf1* Isoform II in naïve cells. Finally, this isoform change by *Tardbp* was confirmed in human CD8^+^ T cells responding to CD3/CD28 costimulation. Specifically, human PBMCs received either Cas9+gRNA control or gRNA against *Tardbp* and were then stimulated with CD3/CD28 beads, cultured for 4 days in IL-2 containing media, and sorted for activated cells expressing CD25. Western blot for Ikzf1 in activated CD8^+^ T cells once again showed a significant decrease in expression of Isoform II in the *Tardbp* KO group when compared with control gRNA, validating that *Tardbp* also controlled protein isoform splicing changes in human cells (Fig. 4f). Both mouse and human Ikzf1 isoform ratio changes were quantified among three independent experiments shown in 4e and f bottom panels. These results indicated that *Tardbp* mediates specific exon changes in TCR signaling genes in activated CD8^+^ T cells.

### *Tardbp* regulates costimulation-specific AS and APA events in CD8^+^ T cells

**Fig. 5:**
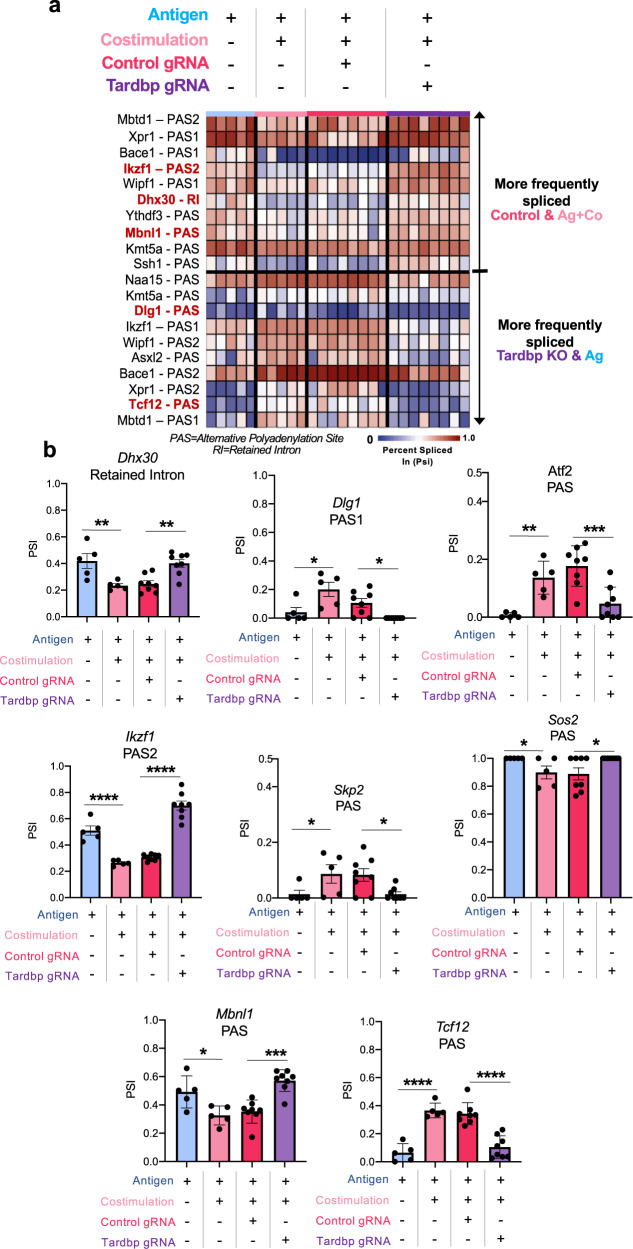
*Tardbp* regulates costimulation-specific alternative splicing and polyadenylation events in CD8^+^ T cells. **a** Deep RNA-seq analyzed with Whippet of Ag, Ag+Co, Control, and Tardbp KO CD8^+^ T cells after 4 days in vivo. Filtering included probability >0.9, Percent-spliced in (Psi) difference > 0.1 or <−0.1; total reads >50; top 20 splicing events of 41 shown by Psi difference, *n* = 5/group for Ag and Ag+Co, *n* = 8/group for Ctrl and Tardbp gRNA. **b** Examples of specific RNA alterations from (**a**) and Supplementary Data 5 (highlighted), *n* = 5/group for Ag and Ag+Co, *n* = 8/group for Ctrl and Tardbp gRNA. PAS = Alternative Polyadenylation Site. RI = Retained Intron. PAS1 or PAS2 indicate the presence of multiple PAS sites in one gene, with PAS1 being the upstream location and PAS2 the downstream. Selection criteria for Fig. 5 is shown in Supplementary Fig. 6. Data are presented as mean values +/− SD using a two-sided t test with Benjamini, Krieger and Yekutieli to control FDR where * represents *n* digits after decimal for *p* value. Source data are provided as a Source Data file.

To address the original hypothesis that costimulation induces *Tardbp* to alter the splicing pattern of CD8^+^ T cells, we selected the alternative splicing and polyadenylation events significantly different between the Ag+Costim versus the Ag group (Fig. 2a) and then filtered these splicing events for *Tardbp* dependency using the control (Ctrl) and *Tardbp* knockout (KO) data in Fig. 4c (Supplementary Fig. 6). Critically, 41 splice events were exclusively costimulation and *Tardbp* dependent; these 41 events were predominantly composed of alternative polyadenylation (APA) changes (Supplementary Data 5). Moreover, 25 of these events were paired polyadenylation sites (PAS)—paired PAS events are indicated as PAS1 or PAS2, referring to the use of an upstream (PAS1) or downstream (PAS2) polyadenylation site within the 3′UTR of the gene (Fig. 5a, top 20 ranked by Psi difference shown). Six genes with splice events (*Ikzf1, Dhx30, Dlg1, Sos2, Atf2, and Skp2*) were previously identified to be induced by costimulation (out of the 26 initially identified), found in T cell costimulation pathways, and observed in *Tardbp* CLIP-seq data seen in Fig. 3 (Figs. 3, 5b and Supplementary Data 5, highlighted). In addition, we found that APA changes to the 3′UTR in the genes *Ikzf1*, *Mbnl1*, and *Tcf12* were identified in two separate analysis of APA using Whippet and DaPars (Fig. 5b). Importantly, these data showed that knockout of *Tardbp* in costimulated CD8^+^ T cells returned splice events to Psi levels seen with Ag alone (Fig. 5a Column 1 (Ag) and Column 4 (*Tardbp* KO)). In total, we found that specific alternative RNA splicing and polyadenylation events are directly dependent on the presence of *Tardbp* during the process of CD8^+^ T cell costimulation. In other words, *Tardbp* controls AS and APA events induced by costimulation of CD8^+^ T cells.

### Optimal effector function of costimulated CD8^+^ T cells requires *Tardbp*

**Fig. 6:**
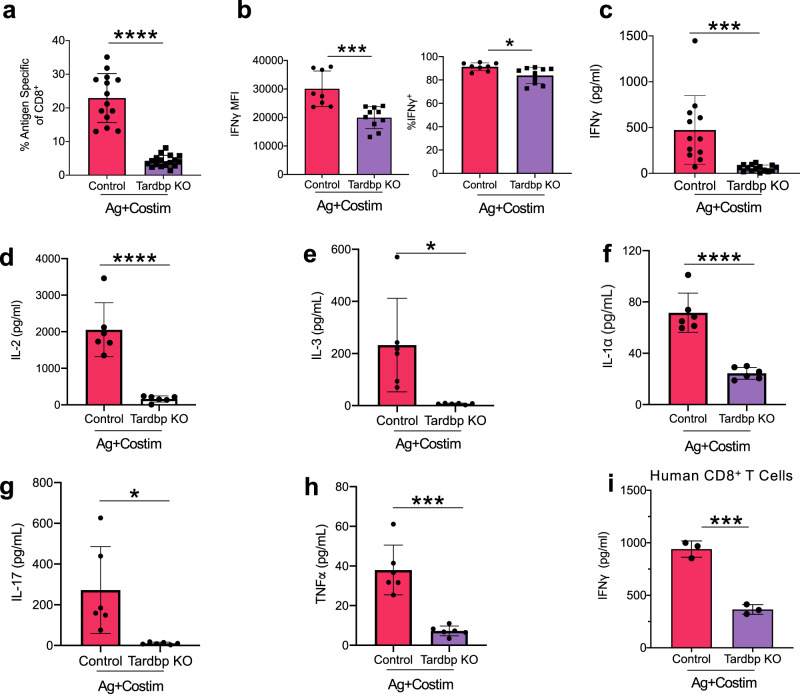
Optimal effector function of costimulated CD8^+^ T cells requires *Tardbp*. **a** Expansion of antigen specific CD8^+^ T cells with and without splicing factor *Tardbp* after costimulation processed as in 4A, *n* = 14 for Ctrl, *n* = 17 for Tardbp KO over 3 independent biological replicates. **b** Intracellular staining for IFNγ after 4 h restimulation with PMAi measured by mean fluorescence intensity (MFI) (left panel) & percentage of Live, TCR-Vα2^+^, TCR-Vβ5^+^, CD45.1^+^, CD8^+^ T cells that are IFNγ^+^ (right panel) between Ctrl and *Tardbp* knockout groups, *n* = 8 for Ctrl, *n* = 10 for Tardbp KO over three independent biological replicates. **c** IFNγ levels by ELISA 20 h after restimulation with PMAI between Ctrl and *Tardbp* KO groups, *n* = 12 for Ctrl, *n* = 15 for Tardbp KO, 3 independent biological replicates. **d**–**h** Inflammatory cytokine levels by multiplex ELISA 20 h after restimulation with PMAI between Ctrl and *Tardbp* KO groups, *n* = 6 per group. **i** IFNγ levels by ELISA 20 h after restimulation with PMAi between Ctrl and *Tardbp* KO groups of human CD8 T cells 4 days after CD3/CD28, n = 3/group, each point is an independent biological replicate. All mouse restimulation experiments were conducted on cells sorted directly ex-vivo. Data are presented as mean values +/− SD using a two-sided t test with Benjamini, Krieger and Yekutieli to control FDR where * represents n digits after decimal for *p* value. Source data are provided as a Source Data file.

Finally, we tested if *Tardbp* was important for CD8^+^ T effector function. It is not feasible to examine *Tardbp’s* function in the absence of costimulation primarily because these cells undergo peripheral deletion in vivo without costimulation^2,3^. Thus, our experiments assessed the requirement of *Tardbp* for CD8^+^ T effector function after costimulation. First, clonal expansion of antigen-specific costimulated CD8^+^ T cells 3.5 days after immunization in the *Tardbp KO* group was significantly decreased compared to control (Fig. 6a). Importantly, clonal expansion levels of CD8^+^ T cells in the *Tardbp KO* group (mean of 4.2%) closely matched that seen after delivery of antigen alone (mean of 2.8%) (Fig. 1a vs. Fig. 6a). Second, after ex-vivo restimulation of antigen-specific CD8^+^ T cells, mean fluorescence intensity (MFI) of IFNγ was robustly reduced in *Tardbp* KO cells, indicating their ability to produce IFNγ was intact but not optimal (Fig. 6b, left panel). Importantly, 91.3% of antigen-specific CD8^+^ T cells were IFNγ+ in control samples and 83.9% were IFNγ+ positive in Tardbp KO samples, indicating the ability to still produce IFNγ by Tardbp KO cells albeit as a much lower level (Fig. 6b, right panel). Third, ex-vivo restimulation of *Tardbp* KO CD8^+^ T cells had significantly decreased secretion of IFNγ, IL-2, TNFα, IL-17, IL-3, and IL-1α (Fig. 6c–h). The reduction in expansion and cytokines highlighted *Tardbp* as essential for optimal effector function of CD8^+^ T cells. Importantly, we tested whether human CD8^+^ T cells responding to CD3/CD28 costimulation were also dependent on splice factor *Tardbp* for optimal effector function. ELISA after restimulation showed a significant decrease of IFNγ levels in *Tardbp* KO human CD8^+^ T cells (Fig. 6i). In total, in vivo and in vitro data show that *Tardbp* is an essential regulator of alternative RNA splicing and polyadenylation, consequential protein isoform changes, clonal expansion, and optimal effector function of CD8^+^ T cells receiving costimulation from mice and humans. Finally, these data point to a mechanism of CD8^+^ T cell costimulation that directly depends on RBPs regulating alternative splicing and polyadenylation to optimize clonal expansion and effector function during an antigen-specific immune response.

## Discussion

The accepted understanding of T cell activation has offered that costimulation acts through increased transcription to regulate clonal expansion, inflammatory function, and survival of T cells^1^. Although transcription is required for CD8^+^ T cell activation, we demonstrate that alternative splicing and alternative polyadenylation changes explain the actions of costimulation and regulate optimal T effector function. This conclusion hinges on the following findings seen here: (1) Costimulation (but not priming alone) induces significant upregulation of RNA binding proteins involved in AS and APA. (2) Costimulation induces hundreds of AS and APA changes in CD8^+^ T cells, (3) RNA binding protein *Tardbp* controls AS and APA events in CD8^+^ T cells, and (4) *Tardbp* is necessary for optimal CD8^+^ T effector function after costimulation. AS and APA changes have been previously shown during T cell activation^40^. However, few large-scale studies have conclusively demonstrated the necessity of RNA binding proteins regulating AS and APA for optimal T cell function after activation in vivo. Thus, regulation of costimulation via AS and APA events controlled by RNA binding proteins such as *Tardbp* represents a mechanism necessary for effector CD8^+^ T cell function.

Conventional wisdom highlights that antigen recognition by the T cell receptor induces the synthesis of the transcription factors NFAT, AP-1, and NFkB^41^. Further, costimulation increases the production of the AP-1 and NFkB transcription factors, thereby increasing the transcription of IL-2 mRNA^41^. Our data show, that while there are indeed transcriptional changes in NFAT, AP-1, NFkB, and IL-2 transcripts, they primarily occur after antigen priming (Supplementary Fig. 2b). Transcriptional changes after costimulation, however, are dominated by the upregulation of RBPs regulating AS and APA (Supplementary Fig. 2a). While antigen-primed cells are not overtly anergic given their ability to expand and produce IFNγ seen in Fig. 1a and b, the signals of costimulation, carried out by downstream RBPs, optimize response to promote maximal CD8^+^ T cell activation. We selected CD134 and CD137 costimulation in this study as it was previously shown to significantly increase effector potential of T cells and is the subject of a recent cancer immunotherapy clinical trial^42^. The knockout of RBP Tardbp in both CD134/CD137 costimulation and CD28 costimulated CD8^+^ T cells showed a significant decrease in effector function of these cells. It is important to note that CD28 is constitutively expressed on CD8^+^ T cells, while CD134/CD137 signals are specifically induced after TCR activation^43^. Despite this difference, we expect CD28 signaling to induce RBPs and show in Fig. 4f (naïve to costimulated) that these human CD8^+^ T cells have endogenous splicing changes in Ikzf1 and are dependent on Tardbp for activation (Fig. 6i). In total, this evidence suggests that several costimulation signals can induce RBP-mediated AS and APA changes, alongside minor transcriptional changes in canonical signaling genes NFAT, AP-1, and NFkB after priming, to allow for optimal effector function of CD8^+^ T cells.

Importantly, we found that AS events favor core exon splicing, with primarily exon skipping events in costimulated cells. Further, as we and others have shown that there is significant shortening of the 3′UTR of transcripts after costimulation due to APA (Fig. 2e and Supplementary Fig. 4e), it is possible that this leads to altered transcript stability leading to decreased mRNA decay and changes in RBP interactions^44^. When paired with the finding that retained introns were significantly reduced in costimulated cells, this offers a concept suggesting that costimulation increases the efficiency of splicing and alternative polyadenylation. In other words, costimulation-induced increases in expression of RNA binding proteins controlling AS and APA may stimulate greater splicing of transcripts to produce functional and stable mRNA isoforms, resulting in a functional protein. Our supplemental data offers further support for this hypothesis, as costimulated CD8^+^ T cells show an increase in NMD-insensitive transcripts (Supplementary Fig. 4f). This is in contrast to unproductive isoforms that, often, contain intron regions and stop codons inhibiting protein production and leading to NMD-sensitive transcripts. Furthermore, the observation of increased protein domain gain was surprising given the prevalence of exon skipping in costimulated CD8^+^ T cells—however, we attribute these protein consequences to potentially skipping exon and intron regions that target transcripts for NMD (Supplementary Fig. 4d, f). In total, our work offers the hypothesis that, in fact, priming of T cells first increases transcript abundance of immune signaling genes. At this stage, a number of these transcripts are “unproductive” and are targeted for NMD. Subsequent costimulatory signal induces upregulation of RNA binding proteins that carry out AS and APA, specifically increasing exon skipping, decreasing intron retention, and shortening 3′UTRs, to produce protein-coding transcripts necessary for optimal effector T cell response.

Given that we found splicing sites in TCR signaling genes specifically controlled by *Tardbp*, this suggests an essential role for *Tardbp* in controlling TCR signaling gene isoforms in the process of T cell activation (Fig. 5). Further, prior work has shown a role for additional RBPs during T cell activation^40^. As we found 18 other RBPs to be upregulated after costimulation and found binding sites for many RBPs near splicing sites changed after T cell costimulation, it would be of interest to test the role of these RBPs in T cell function and contributions to the actions of costimulation. Recent studies have explored the use of pharmacological modulators of splicing to enhance anti-tumor immunity, though none have done so in T cells^45,46^. A critical application of understanding RBP regulation of T effector function could be, for example, use in CAR-T therapy. Here, transcript-specific splicing modifications with CRISPR/Cas9 could be used to induce a more favorable activation profile of T cells without changing gene levels globally as is done currently by the addition of costimulatory signaling domains. Indeed, the adoptive transfer-CRISPR/Cas9 model used in this study closely mimics the CAR-T cell process in vivo and can be further used to therapeutically test isoform-specific modifications. Modifying essential splicing changes in T cells from patients has recently been explored by the use of targeted anti-sense oligonucleotides^47^. Finally, one major question that remains is whether RNA splicing and/or polyadenylation are sufficient to induce T cell activation. Further studies are needed to elucidate the ability of specific RBPs, AS, and APA to regulate T cell functions. In conclusion, costimulation of CD8^+^ T cells leads to RBP-driven reprogramming of the transcript isoform landscape; a requirement for optimal T effector function.

## Methods

### Mice

All animal studies were performed in accordance with UConn Health (Farmington, CT) Institutional Animal Care and Use Committee regulations and were approved by the committee. Mice were maintained on a 12 h dark/light cycle at 30–70% humidity and 20–26.1 °C. C57BL/6J CD45.2 (WT) mice were purchased from The Jackson Laboratory (Bar Harbor, ME). Ova (SIINFEKL^257–264^)-specific OT-I TCR transgenic, recombination activating 1-deficient (*Rag1*^*−/−*^) or (*Rag2*^*−/−*^) mice that were WT on C57BL/6J CD45.1 or C57BL/6J CD45.1/2 Het background were bred in house (OT-I CD45.1, OT-I CD45.1/CD45.2) or purchased from Taconic (OT-I CD45.2 Model #2334, Rensselaer, NY). All mice were maintained in the UConn Health Animal Facility in accordance with National Institutes of Health guidelines. All mice used in these studies were between 1.5 and 8 months of age including both female and male sex.

### Adoptive transfer and immunizations

Naïve splenic OT-I cells (5 × 10^5^ viable CD8^+^ Vα2^+^ Vβ5^+^ CD45.1^+^, CD45.2^+^, or CD45.1^+^/CD45.2^+^) were intravenously transferred into WT C57BL/6J recipients. As before^2,3^, recipient mice were injected intraperitoneally with 100 µg SIINFEKL^257–264^ peptide (InvivoGen, San Diego, CA) + 100 µg of rat IgG control (Ag group) or with agonist anti-CD134 (Clone OX-86 mAb; Bio X Cell, Lebanon NH) and agonist anti-CD137 (Clone 3H3 mAb; Bio X Cell) (Ag+Costim group) 4–6 h after adoptive transfer. Flow cytometry was used to calculate percentage of CD8^+^ CD45.1^+^, Vα2^+^, Vβ5^+^ of total CD8+ from spleen and lymph nodes as in Fig. 1a prior to sorting. Cells from spleen and lymph nodes were sorted for CD8^+^ CD45.1^+^, Vα2^+^, Vβ5^+^ markers 3 days and 12 h after stimulation by flow cytometry for >95% purity prior to use in downstream assays shown in Fig. 1b–f.

### Flow cytometry

Surface staining: naïve OT-I splenocytes for adoptive transfer were identified as (catalog/clone): LIVE/DEAD Fixable Blue Dead^−^ (ThermoFischer, Catalog #L23105, Waltham, MA), CD8^+^ (BD Biosciences, Catalog #558106, Clone 53–6.7, Franklin Lake, NJ), CD4^−^ (Tonbo Biosciences, Catalog #60–0042, Clone RM4–5, San Diego, CA), CD45.1^+^ (eBioscience, Catalog #17-0453-82, Clone A20, Waltham, MA), CD45.2 (Invitrogen, Catalog# 11-0454-81, Clone 104, Waltham, MA), Vα2^+^ (eBioscience, Catalog #46-5812-80/B20.1), Vβ5^+^ (BD Bioscience, Catalog #553190/MR9-4). Analysis of cells was conducted using LIVE/DEAD Fixable, CD4 (Invitrogen), CD45.1 (eBioscience), CD45.2 (Invitrogen), Vα2 (eBioscience), Vβ5 (BD Bioscience), CD8 (BD Biosciences). LSR Aria IIa (BD Biosciences) was used for acquisition. Viable cell gate is representative of a size gate, single-cell gate, and viability gate. All sorting was done using FACSAria II (BD Biosciences). Gating strategy is shown in Supplementary Fig. 1b.

For intracellular staining^2,3^, cells were incubated for the last 5 h in the presence of GolgiPlug (BD Biosciences), surface stained, fixed with 1.5% PFA, permeabilized with 1% Saponin, and stained at 4 °C overnight with IFNγ (BD PharMingen, Catalog# 554412, Clone XMG1.2, Franklin Lake, NJ). The acquisition was performed by LSRIIa.

Human PBMCs were identified: CD8^+^ (BD Biosciences, Catalog #341051, Clone SK1, Franklin Lake, New Jersey), CD4^−^ (BD Biosciences, Catalog #561840, Clone RPA-T4,Franklin Lake, New Jersey), CD25^+^ (BD Biosciences, Catalog #565106, Clone 2A3, Franklin Lake, New Jersey).

All flow cytometry data were gathered using BD FACSDiva 9.0 and analyzed with FlowJo 10.6.1 (Tree Star, Ashland, OR).

### ELISA

All cells were restimulated for 20 h with phorbol 12-myristate 13-acetate (PMA; 50 ng/ml Calbiochem, Darmstadt, Germany) + ionomycin (1 µg/ml Invitrogen) prior to ELISA. Secreted IFNγ from purified human CD8^+^ T cell culture supernatants was analyzed by ELISA (R&D Systems, Catalog #DIF50C, Minneapolis, MN). Secrete IFNγ from purified mouse CD8^+^ T cell culture supernatants was analyzed by ELISA (R&D Systems, Catalog# MIF00). Multiplex on purified mouse CD8^+^ T cell cultures was conducted in house.

### Bulk RNA-seq

For each group, 10^4^–10^6^ cells were lysed in Trizol lysis buffer. RNA was extracted and processed using a QIAGEN miRNEasy Extraction kit. RNA library construction was done using a SMART-Seq v.4 Ultra-low input kit (Takara Bio, Shiga, Japan) and cDNA was converted to sequencing library using NexteraXT DNA Library Prep Kit with indexing primers (Illumina, San Diego, CA). For transcriptomics, libraries were sequenced for single end 1 × 100bp reads at 30million reads/sample. For deep-sequencing, libraries were sequenced for paired end 2 × 100bp or 2 × 150 bp reads for 50–100 million total reads/sample on a NOVASeq 6000 (Illumina). Quality controlled reads (fastq) were aligned to mouse genome (mm10) using HISAT2 and BAM file conversion, sorting, and indexing were done with Samtools^48,49^. Read counts were obtained from resulting BAM files using Stringtie^50^. For transcriptomics analysis, differentially expressed genes were identified using DESeq2 (FDR < 0.05)^51^. FPKM values from Stringtie of most differentially expressed transcripts by FDR were used to create heatmaps of significant DEGs. Pathway analysis was conducted using PathfindR^52^. Library preparation and sequencing was conducted by the Whitehead Institute Genome Technology Core (Boston, MA).

For RNA splicing analysis, raw reads (fastq) were processed using Whippet for alternative splicing, alternative polyadenylation, or alternative TSS events^29^. Significantly differentially spliced events were identified using probability > 0.9 and percent spliced in (PSI) differential of >0.05 or <−0.05 for deep sequencing of Ag vs Ag+Costim groups and probability >0.9, Psi difference >0.1 or <−0.1, and Total Reads >50 for deep sequencing of CRISPR control gRNA vs Tardbp KO groups.

For additional analysis of RNA splicing and protein isoform changes, read counts obtained from Stringtie were processed using IsoformSwitchAnalyzeR^30^. Significant splice switch changes or protein consequences of isoforms were identified using FDR < 0.05 and dIF > 0.1 or dIF < −0.1.

For additional analysis of alternative polyadenylation sites, aligned BAM files were converted to Bedgraph format and processed using DaPars^31^. Significant polyadenylation events were identified by FDR < 0.05 and Percentage of Distal PolyA site Usage Index (PDUI) > 0.15 or <−0.15.

### Human PBMC stimulation

PBMCs from healthy male and female donors between 18 and 55 years of age (Stem Cell Technologies, Catalog #70025, Vancouver, Canada; informed consent: “This consent form provides information about the research study. You will be asked to read this consent form and the study staff will review the consent form with you and answer any questions you may have. If you decide to participate in the study, you will be asked to sign and date this consent form. You will be given a copy of this signed and dated consent form. You should not join this research study until all of your questions are answered.”) were obtained from Stem Cell Technologies under a protocol approved by the Western Institutional Review Board (WIRB). PBMCs were cultured at 1 × 10^6^ cells/ml in Complete T cell Media (RPMI 1640 (ATCC, Catalog #30–2001, Manassas, VA) 2 mM L-glutamine, 10 mM HEPES, 1 mM sodium pyruvate, 4500 mg/L glucose, and 1500 mg/L sodium bicarbonate, 1× Antibiotic-antimycotic (Gibco, Catalog #15240062, Waltham, MA), 1× Non-essential amino acids (Gibco, Catalog #11140050) and 10 ng/mL recombinant human IL-2 (R & D Systems, Catalog #202-IL-010). Cells were stimulated with anti-CD3/anti-CD28 Dynabeads (Gibco, Catalog #11131D) for 4 days at a 1:1 ratio of cells:beads. Cells were restimulated for 20 h with phorbol 12-myristate 13-acetate (PMA; 50 ng/ml Calbiochem, Darmstadt, Germany) + ionomycin (1 µg/ml Invitrogen).

### Statistics

Unless otherwise indicated, dots represent an individual biological replicate, and summary graphs represent means ± SD. Analyses were performed with GraphPad Prism version 8 (GraphPad Software). For comparisons between groups, we used two sided unpaired t-test. *P* values of <0.05 were considered statistically significant and are further defined as indicated.

### CRISPR/Cas9 in vivo

CRISPR/Cas9 experiments in vivo followed the protocol as previously described by Nussing et al.^22^. Briefly, splenocytes from naïve CD45.1^+^, CD45.2^+^ OT-I mice were enriched for CD8^+^ T cells and separated into 5-10million cells per group. Control gRNA (gcacuaccagagcuaacuca) or gRNA against *Tardbp* (guuuguuggacguuguacag, PAM sequence added by Synthego, Menlo Park, CA) were complexed with Cas9 enzyme and electroporated into CD8^+^ OT-I cells. After recovery, cells were adoptively transferred into C57BL/6J mice. Recipient mice were injected intraperitoneally with 100 µg SIINFEKL^257–264^ peptide (InvivoGen, San Diego, CA) + 100 µg of rat IgG control or agonist anti-CD134 (Clone OX-86 mAb; Bio X Cell, Lebanon, NH) and agonist anti-CD137 (Clone 3H3 mAb; Bio X Cell, Lebanon, NH) 4–6 h after adoptive transfer. Cells were sorted for CD8^+^ CD45.1^+^, CD45.2^+^ markers 3 days and 12 h after stimulation by flow cytometry for >95% purity prior to use in downstream assays. Viable cell gate is representative of a size gate, single-cell gate, and LIVE/Dead negative gate.

### CRISPR/Cas9 in vitro

CRISPR/Cas9 experiments in vitro followed the protocol as previously described by Seki and Rutz^53^. PBMCs from healthy donors were cultured in Complete T cell media and electroporated with Cas9/ control gRNA (gcacuaccagagcuaacuca) or *Tardbp* gRNA (gacgcaaguaccuuagaauu, PAM sequence added by Synthego, Menlo Park, CA) complex. Cells were then stimulated with CD3/CD28 beads for 4 days as described above. CD8^+^ CD25^+^ Live cells were then sorted using FACS for >95% purity prior to use in downstream assays. Viable cell gate is representative of a size gate, single-cell gate, and LIVE/Dead negative gate.

### CLIP-Seq analysis

POSTAR3 database was used for all CLIP-seq binding site analysis^36^. Human CLIP-seq data against RNA binding proteins of interest was visualized using Genome Browser and searched for binding sites within 100 bp of splice site of interest by chromosomal position.

### Mass spectrometry

The fractions of interest were lyophilized, resuspended in trypsin digestion buffer, peptides were obtained and identified by LC MS/MS. Mass Spectrometry was conducted at the UConn Proteomic and Metabolomics Core Facility (Storrs, CT).

### Immunoblotting

Immunoblotting was conducted as described previously^54^. In brief, Protein samples were resuspended in denaturing SDS sample buffer and resolved by SDS-PAGE and immunoblotted. Briefly, samples were resolved on 4–15% SDS PAGE, transferred onto 0.2 μm nitrocellulose membrane (BIORAD, Hercules, CA), probed with primary antibodies, washed, then incubated with secondary HRP-conjugated antibodies or avidin-HRP. Western blot detection was performed using ECL plus (Amersham, Arlington Heights, IL). Antibodies used include: Rabbit anti-Tardbp (Abcam Inc, Clone EPR5810, Catalog #ab109535, 1:1000), Rabbit anti-Ikaros (Cell Signaling Technology, Catalog #14859, Clone D6N9Y, 1:1000), Rabbit anti-Actin (20–33) (Sigma-Aldrich, Catalog #A5060, 1:1000), Goat anti-Rabbit IgG (H+L) Cross-Adsorbed Secondary Antibody HRP (ThermoFisher Scientific, Polyclonal, Catalog #G-21234, 1:5000).

### Reporting summary

Further information on research design is available in the Nature Research Reporting Summary linked to this article.

## Supplementary information

Supplementary Information

Description of Additional Supplementary Files

Supplementary Data 1

Supplementary Data 2

Supplementary Data 3

Supplementary Data 4

Supplementary Data 5

Reporting Summary

## Data Availability

All novel RNA-seq data have been deposited in the Gene Expression Omnibus under accession number GSE200240 at. Source data are provided with this paper.

## Source data

Source Data
