# Supplementary material for: Optimal CD8+ T cell effector function requires costimulation-induced RNA-binding proteins that reprogram the transcript isoform landscape
Source: Nat Commun. 2022 Jun 20;13:3540. doi: 10.1038/s41467-022-31228-0 (PMC9209503; doi:10.1038/s41467-022-31228-0)
Supplement: Supplementary file 1 — Supplementary Information [file 41467_2022_31228_MOESM1_ESM.pdf]

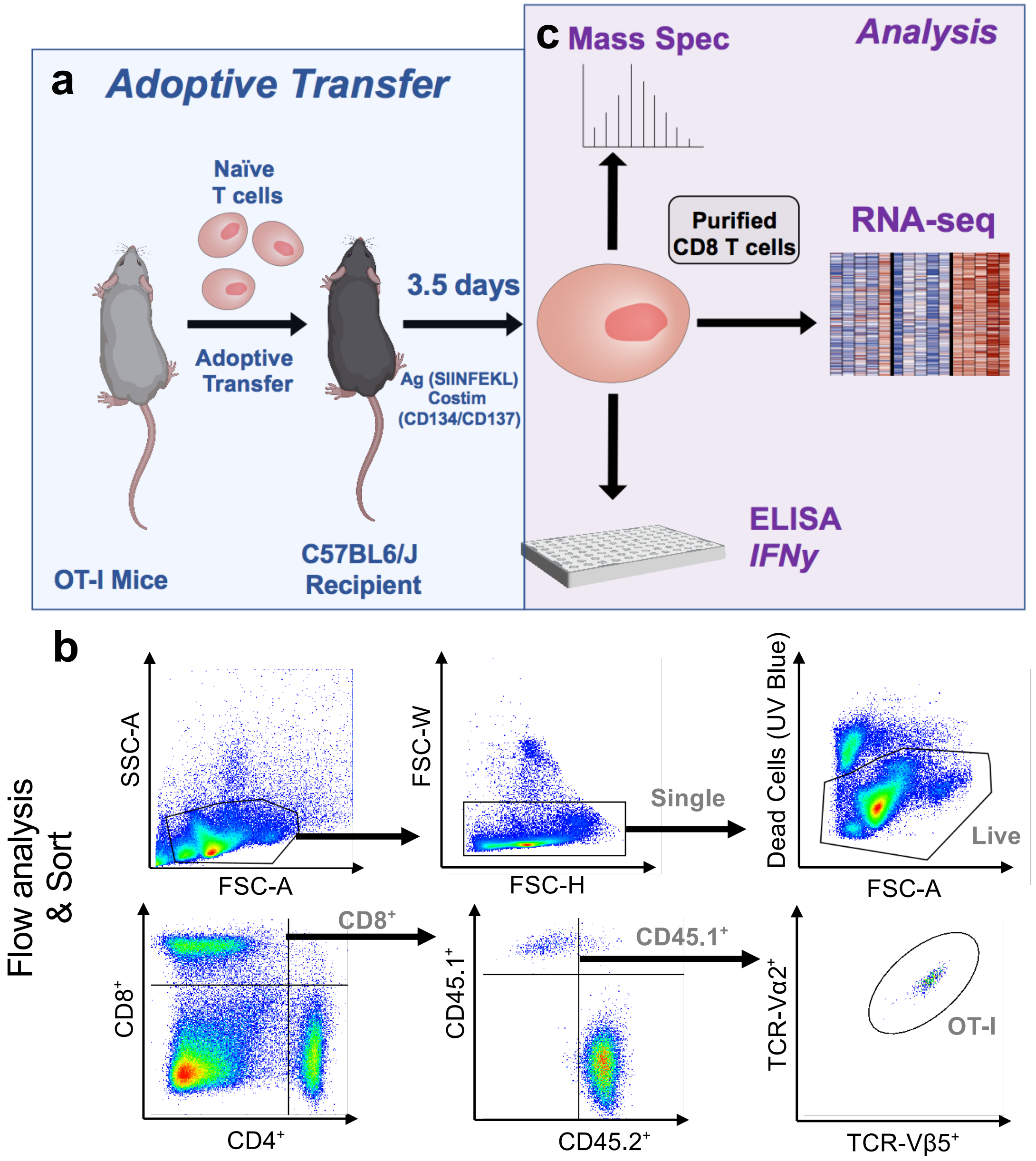

Sup Fig. 1 Representative experimental design for analysis and sort of costimulated CD8⁺ T cells in Fig 1

a. Total splenocytes and lymph nodes were collected from naïve OT-I mice and adoptively transferred into C57BL6/J recipients. Recipients were then immunized with antigen alone (SIINFEKL) or antigen+costimulation (SIINFEKL + anti-CD134 + anti-CD137). Splenocytes and lymph nodes were harvested 3.5 days after adoptive transfer and analyzed for percent TCR-Vα2$^+$, TCR-Vβ5$^+$, CD45.1$^+$ cells of total CD8$^+$ T cell. Splenocytes and lymphocytes from lymph nodes were then sorted for Live, TCR-Vα2, TCR-Vβ5, CD45.1$^+$, CD8$^+$ T cells. **b.** Representative gating strategy of calculation for Fig 1b and sorting for pure TCR-Vα2$^+$, TCR-Vβ5$^+$, CD45.1$^+$, CD8$^+$ T cells. **c.** RNA was extracted for RNA-seq and protein fractionation was performed for mass spectrometry. Sorted, pure CD8$^+$ T cells were restimulated with PMAi for 20h before measurement of IFNγ by ELISA.

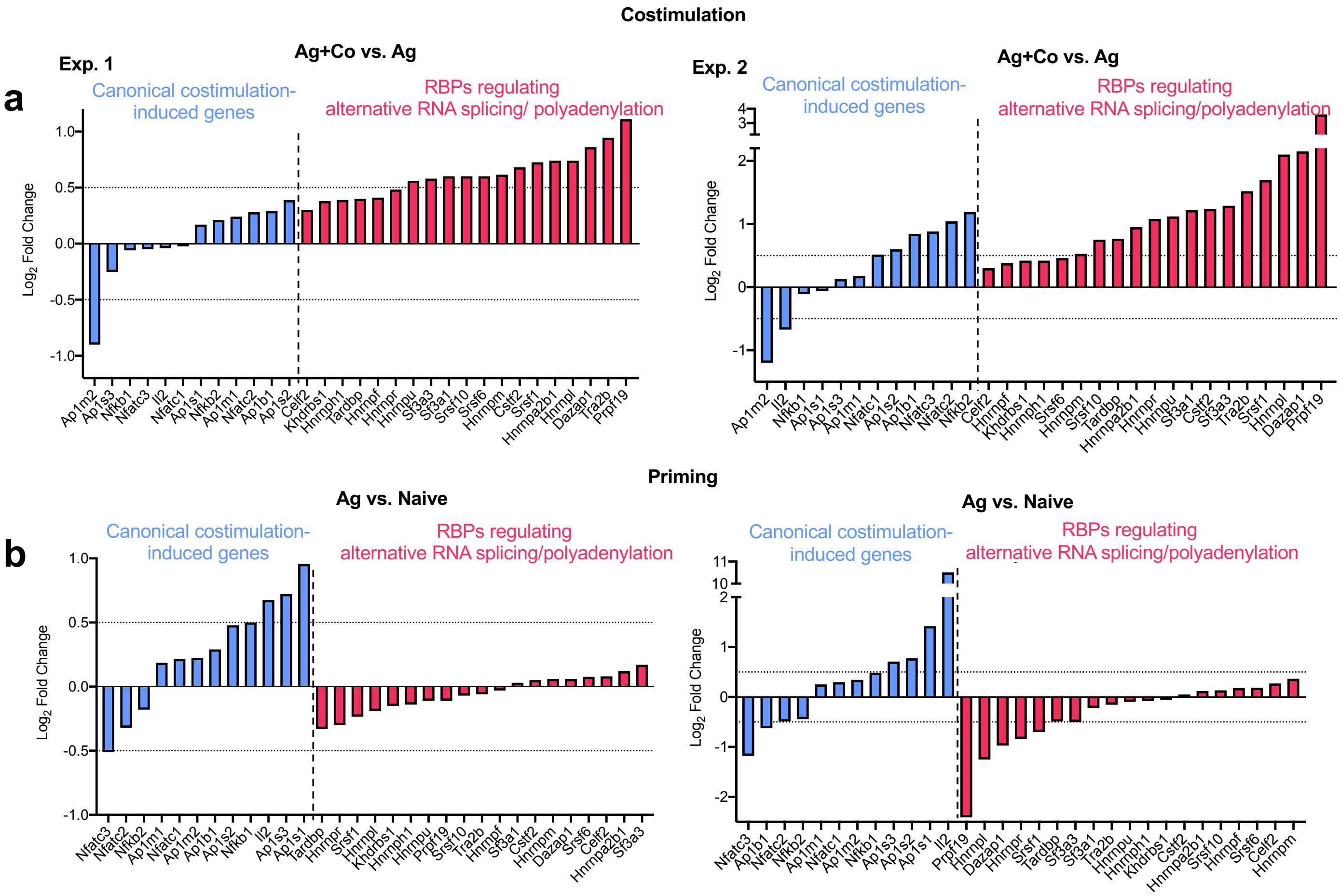

Sup Fig 2: Costimulation of CD8⁺ T cells significantly increases transcript levels of RNA binding proteins

a. Log_2 fold change in transcript of canonical costimulation-induced genes and RBPs regulating alternative RNA splicing found enriched in Fig. 1g compared between costimulated (Ag+Co) and antigen primed (Ag) groups, n=5/group. Data is derived from RNA-seq of cells seen in Fig 1a (OT-I, CD45.1⁺ cells of CD8⁺ T cells 3.5 days after receiving antigen alone and antigen plus costimulation). All RBPs have an FDR<.05; no filtering was used for canonical costimulation genes. Replicate dataset 2 from Sup Fig 2 is shown in the right panel. **b.** Log_2 fold change in transcript of canonical costimulation-induced genes (NFkB, AP1, NFAT, IL2) and RBPs regulating alternative RNA splicing found enriched in Fig. 1g compared between antigen primed (Ag) and naïve CD8⁺ T cells, n= 5/group. Data is derived from RNA-seq of cells seen in Fig 1a (OT-I, CD45.1⁺ cells of CD8⁺ T cells 3.5 days after receiving antigen alone) and naïve OT-I CD8⁺ T cells. No p value filtering was used for this figure. Replicate dataset 2 from Sup Fig 2 is shown in the right panel.

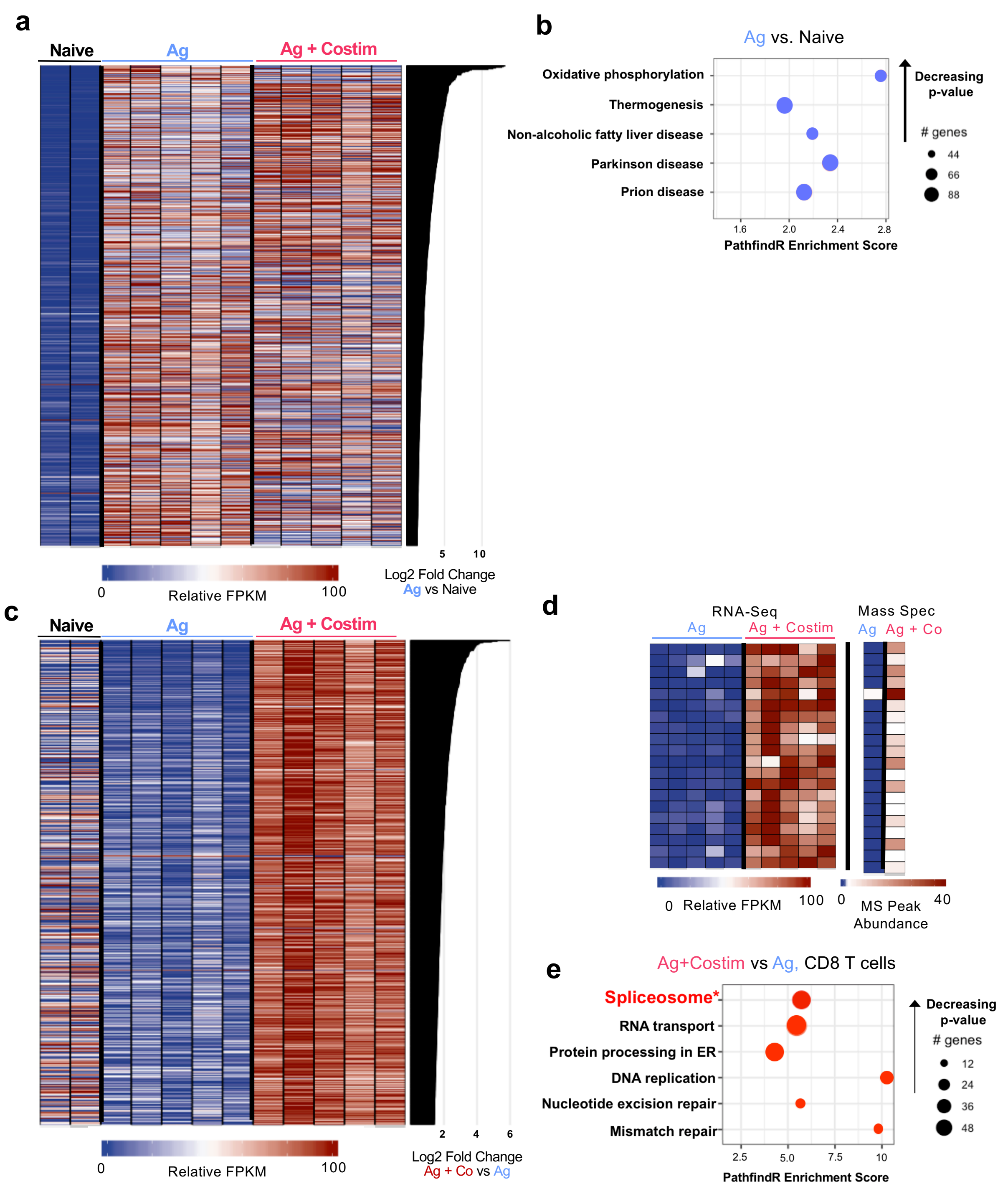

Sup Fig. 3 Costimulation of CD8⁺ T cells induces a unique gene signature of RNA splicing

a. Relative FPKM levels of the top 700 genes ranked by log_2fold change between antigen and naïve groups (FDR<.05). n>2/group. Data is derived from RNA-seq of cells seen in Fig 1a (OT-I, $CD45.1^+$ cells of $CD8^+$ T cells 3.5 days after receiving antigen alone) and naïve OT-I $CD8^+$ T cells. **b**. Pathway analysis of 700 genes ranked by log_2fold change between Ag alone and Naïve (blue). **c.** Relative FPKM levels of 700 genes ranked by log_2fold change (FDR<.05) between Ag+Costim and Ag alone groups, n=5/group. Data is derived from RNA-seq of cells seen in Fig 1a (OT-I, $CD45.1^+$ cells of $CD8^+$ T cells 3.5 days after receiving antigen alone and antigen plus costimulation) **d.** Top 20 (of 925) genes found to be significantly upregulated expressed between Ag+Costim and Ag alone groups in both RNA & mass spectrometry experiments (FDR<.05), n=5/group for RNA, n=1 sample pooled from 5/group for mass spectrometry. **e.** Pathway analysis of 925 genes identified in 1d ranked by log2fold change between Ag+Costim and Ag alone groups.

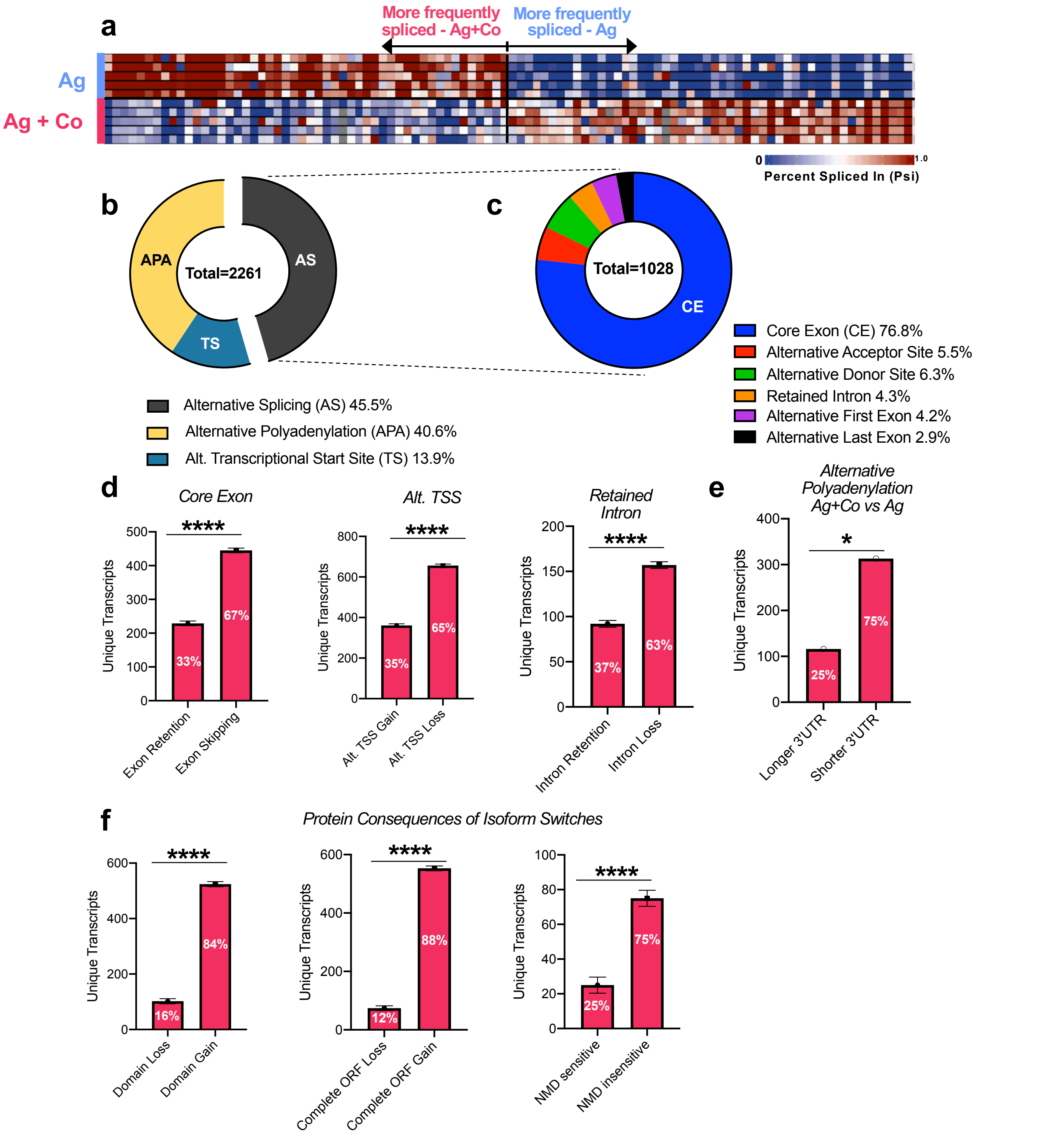

Sup Fig 4: Costimulation of CD8⁺ T cells induces exon skipping, intron loss, and nonsense mediated decay insensitive transcripts

a. Percent spliced in levels (Psi) of 2261 splice site differences in 1000 genes between Ag and Ag+Costim groups (Probability>.9, Psi Diff >.05 or <-.05) after Whippet analysis of RNA-seq of samples from Sup Fig.2 comparing antigen+costim and antigen alone groups (100 shown), n=5/group. **b.** Distribution of RNA alterations of events seen in Sup Fig 3a. **c.** Distribution of alternative splicing events from Sup Fig 3b. **d.** Isoform switch analysis of splicing sites from RNA-seq samples in Sup Fig. 2 showing enrichment of splicing event type between Ag+Costim and Ag groups. Percentages represent a fraction of the total number of exon splicing events between Ag+Costim and Ag groups i.e. 67% of exon splicing events were skipped exons (in the Ag+Co group) while 33% were included exons, n=5 samples/group. **e.** Analysis of alternative polyadenylation sites and 3'UTR length between Ag+Costim and Ag groups, n=5 samples/group. **f.** Predicted protein consequences of isoform switches in Sup Fig 3a. Data are presented as mean values +/- SE derived from two-sided Fisher's exact test with Benjamini-Hochberg adjustment for multiple comparisons, n=5 samples/group.

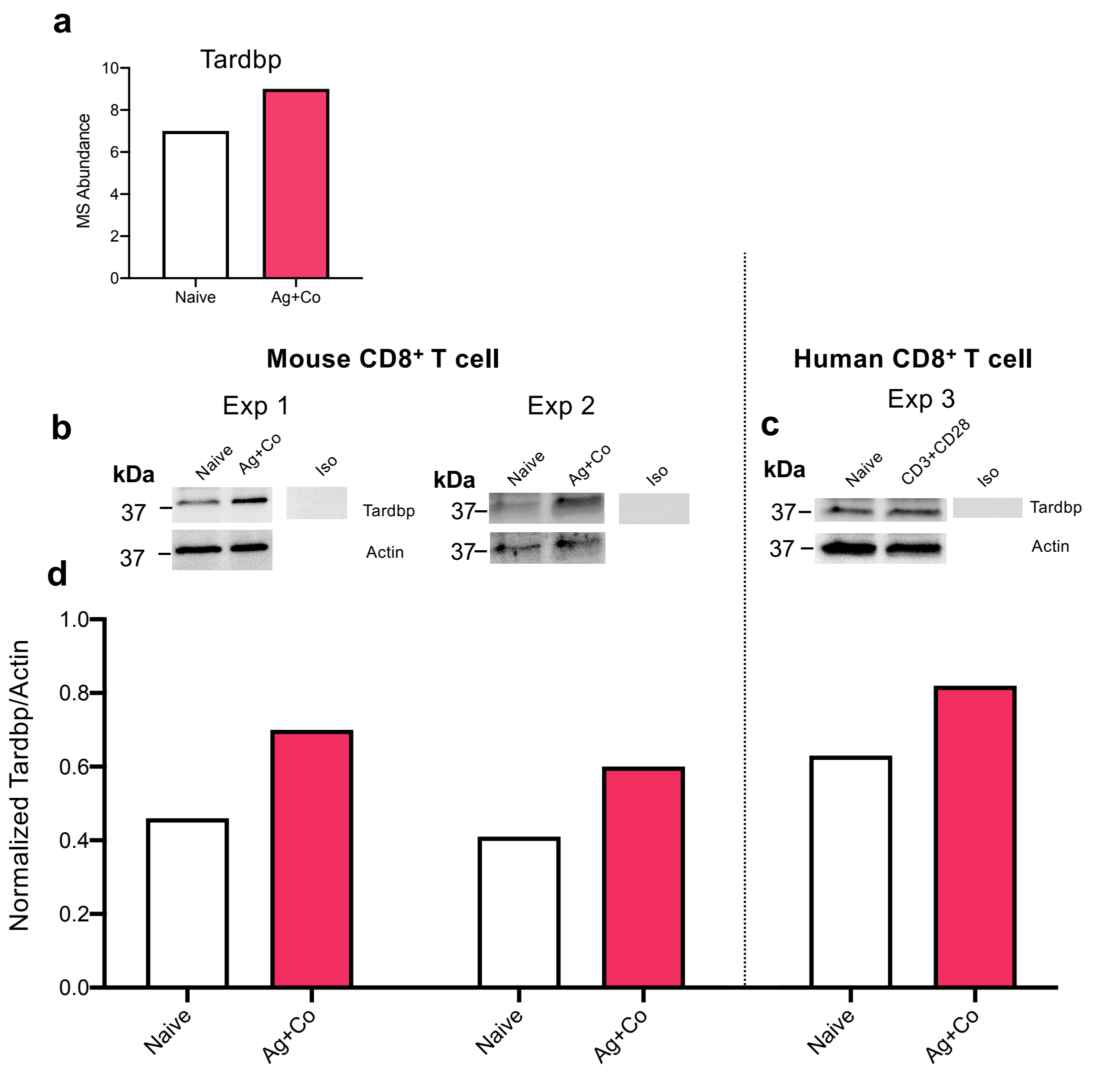

Sup Fig 5: Expression of Tardbp in $CD8^+$ T cell is upregulated by costimulation
a. Tardbp protein levels in cytoplasmic fraction of CD8 T cells receiving no stimulation or Ag+Costim as measured by mass spectrometry. **b.** Immunoblot of Tardbp from $CD8^+$ T cells sorted ex vivo from naïve mice and mice that received antigen and costimulation for 3.5 days (as described in Fig. 1) from 2 independent experiments. **c.** Immunoblot of Tardbp from $CD8^+$ T cells sorted from naïve PBMCs or PBMCs stimulated with CD3/CD28+IL2 from one independent experiment. **d.** Quantification of Tardbp expression normalized to actin comparing antigen + costimulation and naïve groups shown in b and c. A ratio of 1 is equal relative expression between Tardbp and Actin.

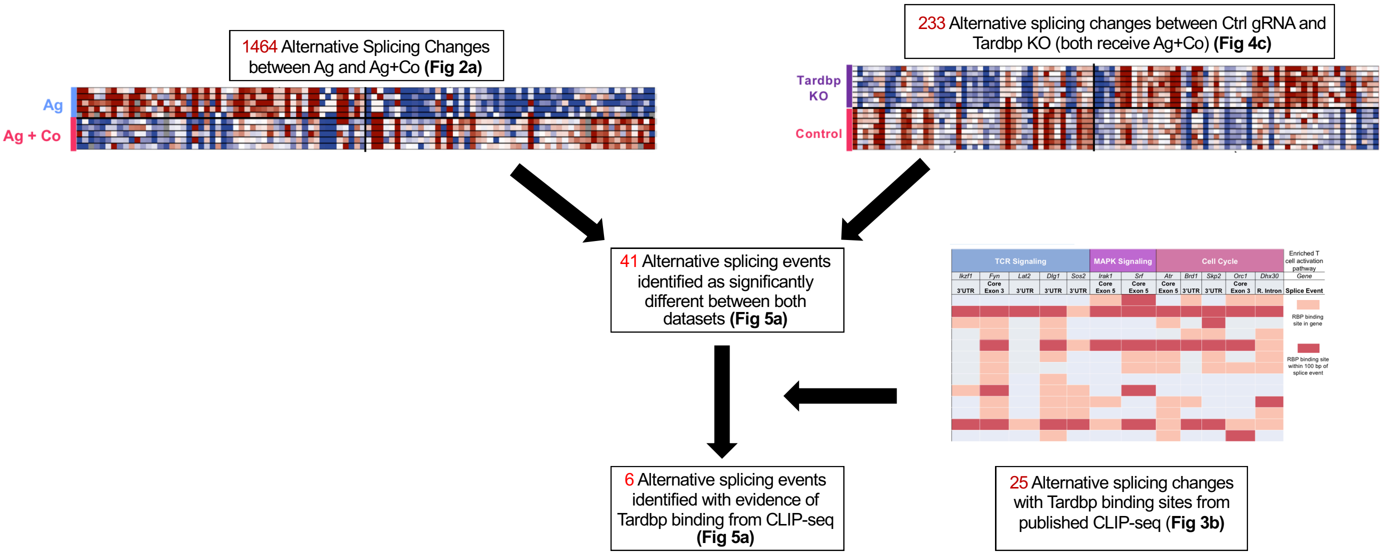

Sup Fig 6. Flow chart of filtering for Fig 5

Fig 5 integrates costimulation-induced splicing events in Fig 2a with Tardbp KO data from Fig 4c as well as Tardbp CLIP-seq binding data in Fig 3b.
