# Supplementary material for: Optimal CD8+ T cell effector function requires costimulation-induced RNA-binding proteins that reprogram the transcript isoform landscape
Source: Nat Commun. 2022 Jun 20;13:3540. doi: 10.1038/s41467-022-31228-0 (PMC9209503; doi:10.1038/s41467-022-31228-0)
Supplement: Supplementary file 2 — Description of Additional Supplementary Files [file 41467_2022_31228_MOESM2_ESM.pdf]

Description of Additional Supplementary Files

Supplementary Data 1: RNA-seq and Mass Spectrometry (MS) of naïve, antigen-primed, and costimulated CD8+ T cells from two independent experiments. Filters include FDR<.05 calculated by DESeq2 using a Negative Binomial Distribution fit and Wald test with subsequence Benjamini-Hochberg adjustment and identification in MS data.

Supplementary Data 2: Percent spliced in (PSI) of RNA regions in antigen-primed and costimulated CD8+ T cells. Filters include PSI>.9 and delta PSI of .05.

Supplementary Data 3: Percent spliced in (PSI) of RNA regions in antigen-primed and costimulated CD8+ T cells repeated. Filters include PSI>.9 and delta PSI of .05.

Supplementary Data 4: Percent spliced in (PSI) of RNA regions in Control and Tardbp KO costimulated CD8+ T cells. Filters include PSI>.9 and delta PSI of .1.

Supplementary Data 5: Percent spliced in (PSI) of RNA regions in Control and Tardbp KO overlapped with PSI of RNA regions found significantly different between antigen-primed and costimulated CD8+ T cells (merging of Supplementary Data 2 and Supplementary Data 4). Filters include PSI>.9 and delta PSI of .05.
