# Supplementary material for: Optimal CD8+ T cell effector function requires costimulation-induced RNA-binding proteins that reprogram the transcript isoform landscape
Source: Nat Commun. 2022 Jun 20;13:3540. doi: 10.1038/s41467-022-31228-0 (PMC9209503; doi:10.1038/s41467-022-31228-0)
Supplement: Supplementary file 8 — Reporting Summary [file 41467_2022_31228_MOESM8_ESM.pdf]

nature portfolio

Corresponding author(s): Anthony Vella, Timofey Karginov

Last updated by author(s): April 7th, 2022

Reporting Summary

Nature Portfolio wishes to improve the reproducibility of the work that we publish. This form provides structure for consistency and transparency in reporting. For further information on Nature Portfolio policies, see our Editorial Policies and the Editorial Policy Checklist.

Statistics

For all statistical analyses, confirm that the following items are present in the figure legend, table legend, main text, or Methods section.

n/a	Confirmed	
☐	☒	The exact sample size (*n*) for each experimental group/condition, given as a discrete number and unit of measurement
☐	☒	A statement on whether measurements were taken from distinct samples or whether the same sample was measured repeatedly
☐	☒	The statistical test(s) used AND whether they are one- or two-sided<br>*Only common tests should be described solely by name; describe more complex techniques in the Methods section.*
☒	☐	A description of all covariates tested
☐	☒	A description of any assumptions or corrections, such as tests of normality and adjustment for multiple comparisons
☐	☒	A full description of the statistical parameters including central tendency (e.g. means) or other basic estimates (e.g. regression coefficient) AND variation (e.g. standard deviation) or associated estimates of uncertainty (e.g. confidence intervals)
☐	☒	For null hypothesis testing, the test statistic (e.g. *F*, *t*, *r*) with confidence intervals, effect sizes, degrees of freedom and *P* value noted<br>*Give P values as exact values whenever suitable.*
☒	☐	For Bayesian analysis, information on the choice of priors and Markov chain Monte Carlo settings
☒	☐	For hierarchical and complex designs, identification of the appropriate level for tests and full reporting of outcomes
☒	☐	Estimates of effect sizes (e.g. Cohen's *d*, Pearson's *r*), indicating how they were calculated

Our web collection on statistics for biologists contains articles on many of the points above.

Software and code

Policy information about availability of computer code

Data collection	BD FACSDiva 9.0 was used to collect FACS experiments
Data analysis	Flow Cytometry data was analyzed using FlowJo 10.6.1. RNA-seq data was analyzed using publicly available software including HISAT2, Samtools, Stringtie, Whippet, DaPars, RStudio 1.2.13 (Bioconductor package, IsoformSwitchAnalyzeR, DESeq2, PathfindR), Microsoft Excel, and Graph Pad Prism v8. Western blot quantification was performed using ImageJ. Peak visualization of CLIP-seq data was done using IGV Browser.

For manuscripts utilizing custom algorithms or software that are central to the research but not yet described in published literature, software must be made available to editors and reviewers. We strongly encourage code deposition in a community repository (e.g. GitHub). See the Nature Portfolio guidelines for submitting code & software for further information.

Data

Policy information about availability of data

All manuscripts must include a data availability statement. This statement should provide the following information, where applicable:

- Accession codes, unique identifiers, or web links for publicly available datasets
- A description of any restrictions on data availability
- For clinical datasets or third party data, please ensure that the statement adheres to our policy

All RNA-seq datasets are publicly available as of April 8th, 2022 at GEO accession GSE200240

Field-specific reporting

Please select the one below that is the best fit for your research. If you are not sure, read the appropriate sections before making your selection.

- [x] Life sciences
- [ ] Behavioural & social sciences
- [ ] Ecological, evolutionary & environmental sciences

For a reference copy of the document with all sections, see nature.com/documents/nr-reporting-summary-flat.pdf

Life sciences study design

All studies must disclose on these points even when the disclosure is negative.

Sample size	The required number of replicates are calculated based on past experience and standards in the field (at least three independent biological replicates for each condition). For in vivo data, this was expanded to at least 8 biological replicates per group. These sample sizes are based on the number of replicates necessary to achieve statistical significance derived from 20 years of publications on these costimulatory receptors
Data exclusions	We did not exclude data from consideration.
Replication	Results were consistently replicated across experiments as indicated in figure legends.
Randomization	No randomization was necessary for this study because we were comparing well-controlled, syngenic mice and from 20 years of publications on these costimulatory receptors randomization is not necessary.
Blinding	Blinding was not needed in the study because conditions were well controlled with syngenic mice - blinding is not typically used in the field and has not been used in 20 years of publications on these costimulatory receptors.

Reporting for specific materials, systems and methods

We require information from authors about some types of materials, experimental systems and methods used in many studies. Here, indicate whether each material, system or method listed is relevant to your study. If you are not sure if a list item applies to your research, read the appropriate section before selecting a response.

Materials & experimental systems

n/a	Involved in the study	
[ ]	[x]	Antibodies
[x]	[ ]	Eukaryotic cell lines
[x]	[ ]	Palaeontology and archaeology
[ ]	[x]	Animals and other organisms
[ ]	[x]	Human research participants
[x]	[ ]	Clinical data
[x]	[ ]	Dual use research of concern

Methods

n/a	Involved in the study	
[x]	[ ]	ChIP-seq
[ ]	[x]	Flow cytometry
[x]	[ ]	MRI-based neuroimaging

Antibodies

Antibodies used	Rabbit anti-Tardbp (Abeam Inc, Clone EPR5810, Catalog #ab109535), Rabbit anti-lkaros (Cell Signaling Technology, Catalog #14859, Clone D6N9Y), Rabbit anti-Actin (20-33) (Sigma-Aldrich, Catalog #A5060), Goat anti-Rabbit IgG (H+L) Cross-Adsorbed Secondary Antibody HRP (ThermoFisher Scientific, Polyclonal, Catalog #G-21234). CDS (Biolegend, Catalog #100712, Clone 53-6.7, San Diego, CA), CD4(Biolegend, Catalog #100566, Clone RM4-5, San Diego, CA), CD45.1 (lnvitrogen, Catalog #25-0453-82, Clone A20, Waltham, MA), CD45.2 (BD Pharmingen, Catalog#561874, Clone 104, Franklin Lake, NJ), Va2 (eBioscience, Catalog #46-5812-80, CloneB20.1), VB5 (BD Biosciences, Catalog #553190, MR9-4,Franklin Lake, New Jersey), IFN (Tonbo Biosciences, Catalog# 75-7311-UlO0, Clone XMGl.2, San Diego, CA). For human PBMCs - CDS (BD Biosciences, Catalog #341051, Clone SKl, Franklin Lake, New Jersey), CD4 (BD Biosciences, Catalog #561840, Clone RPAT4, Franklin Lake, New Jersey), CD25 (BD Biosciences, Catalog #565106, Clone 2A3, Franklin Lake, New Jersey)
Validation	Rabbit anti-Tardbp - Abeam - "Knockout validated"<br>Rabbit anti-lkaros - Cell Signaling Technology - "Validated in 6 different peer-reviewed studies"<br>Goat anti-Rabbit IgG (H+L) Cross-Adsorbed Secondary Antibody HRP - ThermoFisher Scientific - 631 publications referenced<br>anti-mouse Actin (20-33) - Millipore Sigma - "Validated in over 590 articles"<br>anti-mouse CDS - Biolegend - "FC-quality tested" - 12 publications referenced<br>anti-mouse CD4 - Biolegend - "FC-quality tested" - 12 publications referenced<br>anti-mouse CD45.l - Invitrogen - 76 publications referenced<br>anti-mouse CD45.2 - BD Pharmingen - 9 publications referenced<br>anti-mouse V 2 - eBioscience - 2 publications referenced<br>anti-mouse V 5 - BD Bioscience - 9 publications referenced

	anti-mouse IFN - Tonbo Biosciences - 10 publications referenced<br>anti-human CDS - BD Biosciences - 24 publications referenced<br>anti-human CD4 - BD Biosciences - 2 publications referenced<br>anti-human CD25 - BD Biosciences - 13 publications referenced

Animals and other organisms

Policy information about studies involving animals; ARRIVE guidelines recommended for reporting animal research

Laboratory animals	C57BL/6J CD45.2 recipients (Jackson Laboratory, #000664, Bar Harbor, ME), B6.129S6-Rag2tm1Fwa Tg(TcraTcrb)Il00Mjb (Taconic Biosciences, #2334, Rensselaer, NY), both females and males were use in the study, 6-12 weeks of age. Mice were maintained on a 12 hour dark/light cycle at 30-70% humidity and 20-26.1 degrees C
Wild animals	Study did not involve wild animals
Field-collected samples	Study did not involve samples collected from the field
Ethics oversight	All animal studies were performed in accordance with UConn Health (Farmington, CT) Institutional Animal Care and Use Committee regulations and were approved by the committee.

Note that full information on the approval of the study protocol must also be provided in the manuscript.

Human research participants

Policy information about studies involving human research participants

Population characteristics	Inclusion criteria: Age range: 18-55 years<br>• Weight: 120 lbs minimum<br>• Negative test for infectious diseases such as HIV type I and II; Hepatitis B and C<br>• Platelets: 130,000-400,000 cells/mcL<br>• Hemoglobin of 13.0 g/dl and greater for males, and 12.0 g/dl and greater for females<br>• Pulse rate of 50-100 bpm<br>• Mental competence to understand info1med consent fonn<br>• Considered to be in good general health as detennined by enrollment process<br>• US citizen<br>• Subjects that have signed the ICF
Recruitment	Cells are obtained by STEMCELL Inc. from donors who are voluntarily participating in a donor program approved by an IRB by the Western Institutional Review Board (WIRB) and have either donated their cells or have been reasonably compensated for their time and effort during their donation, as approved by an IRB.
Ethics oversight	STEMCELL collects donor demographic information ethically, using consent forms and protocols approved by either an Institutional Review Board (IRB) Western Institutional Review Board (WIRB). Informed Consent Statement (full IRB study approval provided on request): This consent form<br>provides information about the research study. You will be asked to read this consent form<br>and the study staff will review the consent form with you and answer any questions you may<br>have. If you decide to participate in the study, you will be asked to sign and date this<br>consent form. You will be given a copy of this signed and dated consent form. You should<br>not join this research study until all of your questions are answered.

Note that full information on the approval of the study protocol must also be provided in the manuscript.

Flow Cytometry

Plots

Confirm that:

- [x] The axis labels state the marker and fluorochrome used (e.g. CD4-FITC).
- [x] The axis scales are clearly visible. Include numbers along axes only for bottom left plot of group (a 'group' is an analysis of identical markers).
- [x] All plots are contour plots with outliers or pseudocolor plots.
- [x] A numerical value for number of cells or percentage (with statistics) is provided.

Methodology

Sample preparation	Splenocytes and lymph nodes were collected, red blood cells were lysed, and samples were stained with antibody preparations. For human commercially available PBMCs, cells were washed and stained with antibody preparations.
Instrument	LSR Aria IIa, LSRII

Software	BD FACSDiva 9.0 , FlowJo 10.6.1
Cell population abundance	Live, CD8+, CD45.l +/CD45.2+ cells were sorted for >95% purity. For data collection purity for antigen-specific T cells was verified at >95% of same sorted samples using TCR-specific V 2+,V 5+ gate. For human PBMCs, cells were sorted for Live, CD8+, CD25+ for >95% purity.
Gating strategy	Cells were gated for a lymphocyte SSC-A, FSC-A gate, single cells by FSC-H, FSC-W, SSC-H,-SSC-W, Live cells by Live-Dead,CD8 +, CD45.l +/CD45.2+, V 2+,V 5+. For human PBMCs, cells were gated by SSC-A, FSC-A gate,single cells by FSC-H, FSC-W, SSCH,-SSC-W, Live cells by Live-Dead,CD8+,CD25+.

☒ Tick this box to confirm that a figure exemplifying the gating strategy is provided in the Supplementary Information.
