# Supplementary material for: Optimal CD8+ T cell effector function requires costimulation-induced RNA-binding proteins that reprogram the transcript isoform landscape
Source: Nat Commun. 2022 Jun 20;13:3540. doi: 10.1038/s41467-022-31228-0 (PMC9209503; doi:10.1038/s41467-022-31228-0)

Immunoblot – Sup Fig 5b, Fig 4e

Anti-mouse Actin

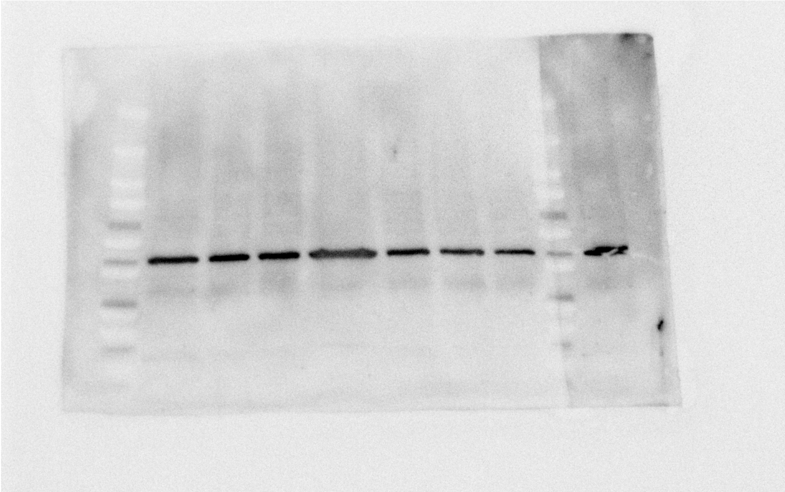

Anti-mouse Tardbp

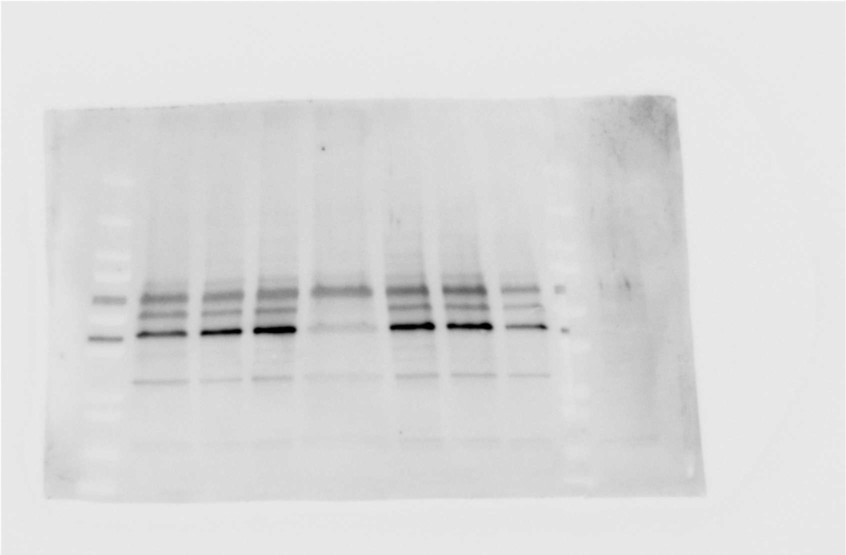

Anti-mouse Ikzf1

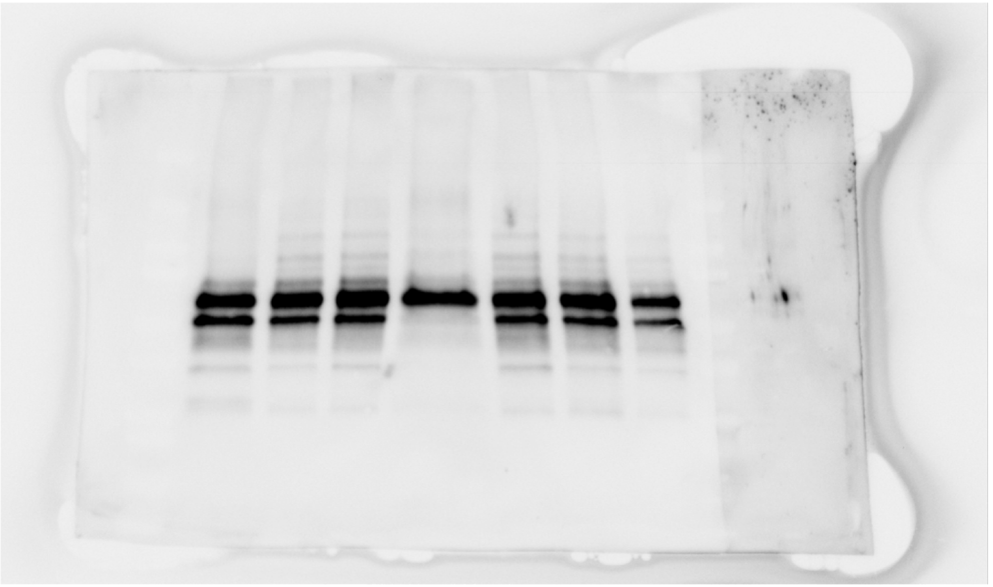

Immunoblot – Sup Fig 5c

Anti-human Actin

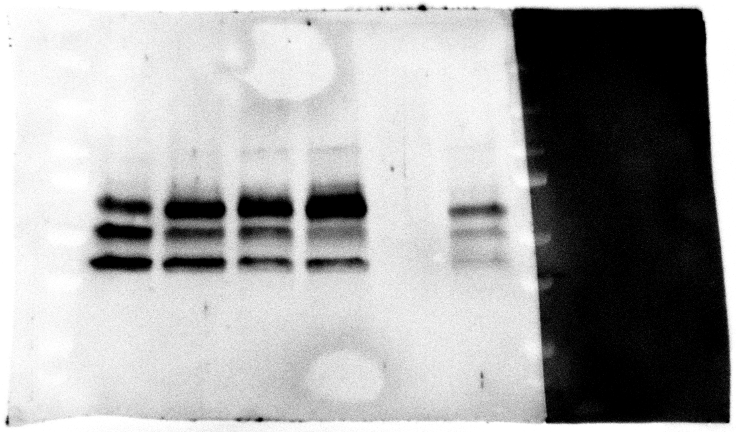

Anti-human Tardbp

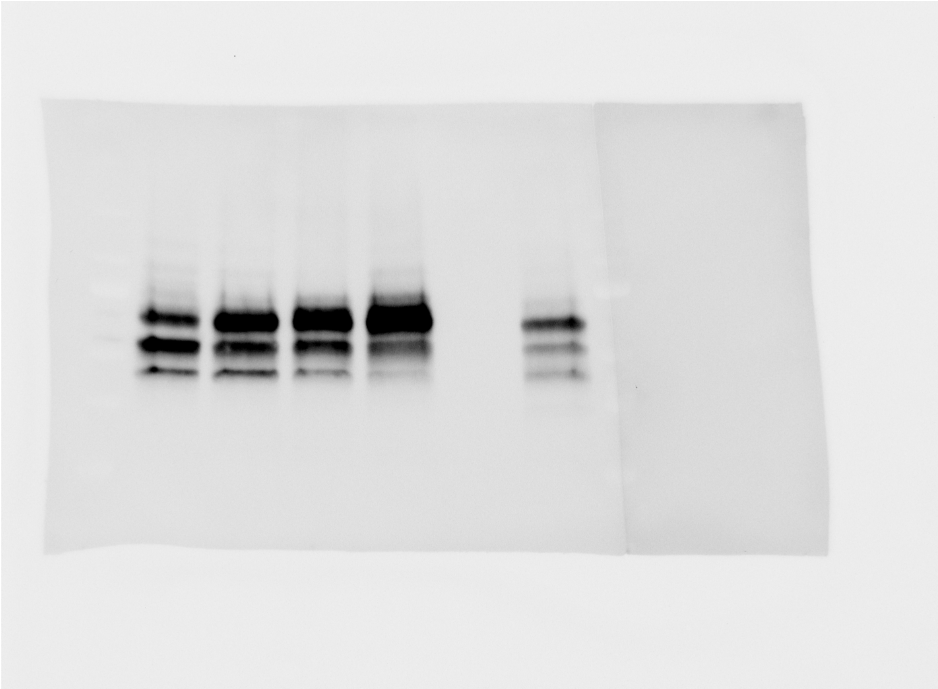

Immunoblot – Fig 4f

Anti-human Ikzf1

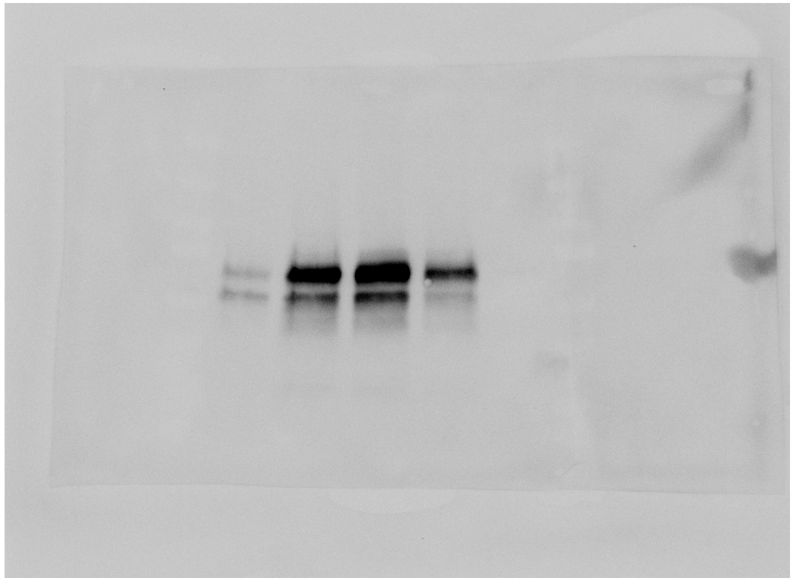

Anti-human Tardbp

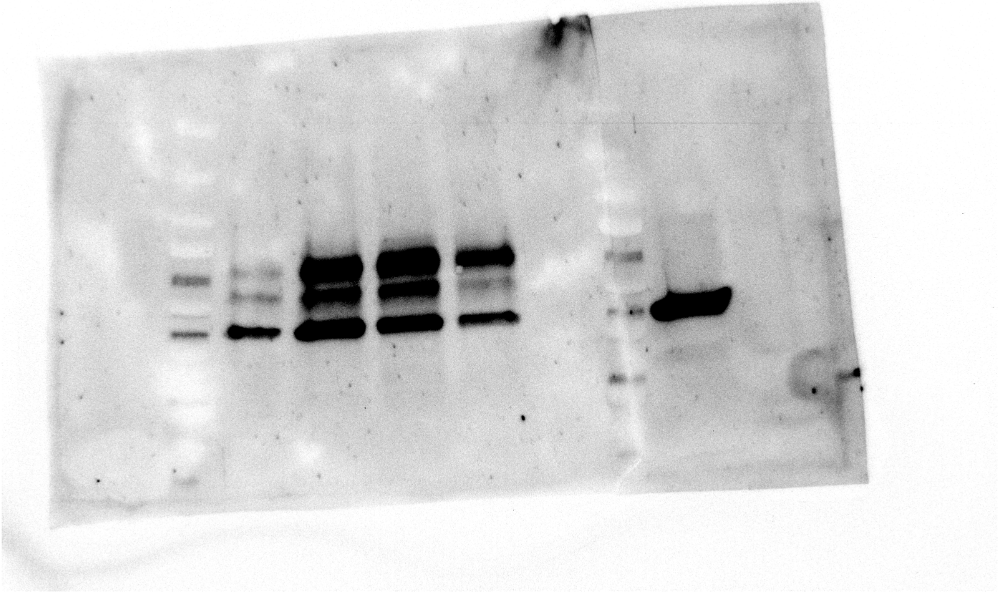

Anti-human Actin

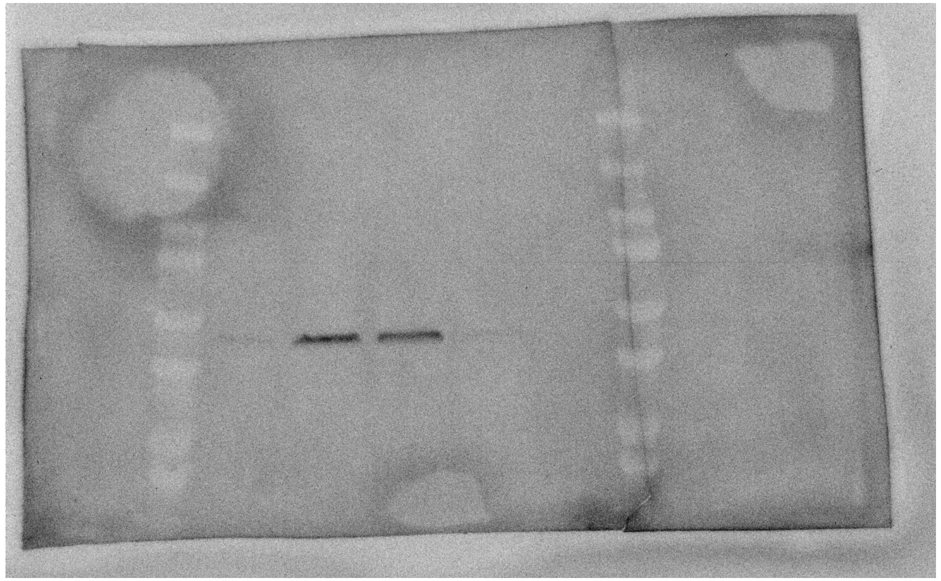

Immunoblot – Sup Fig 5b

Anti-mouse Actin

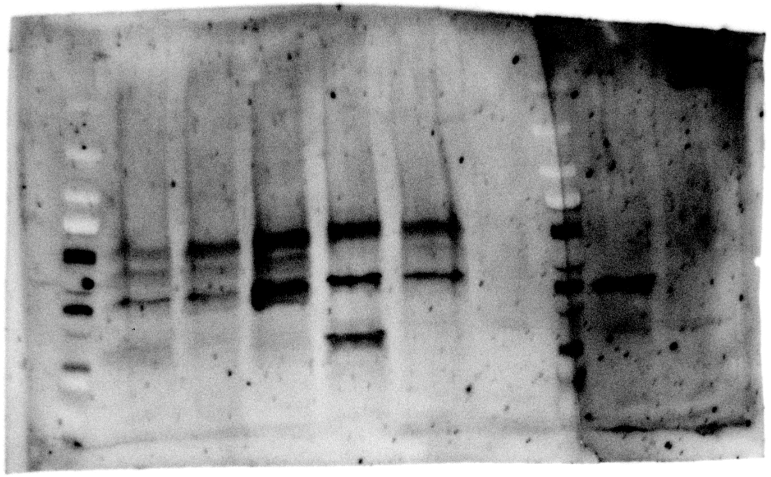

Anti-mouse Tardbp

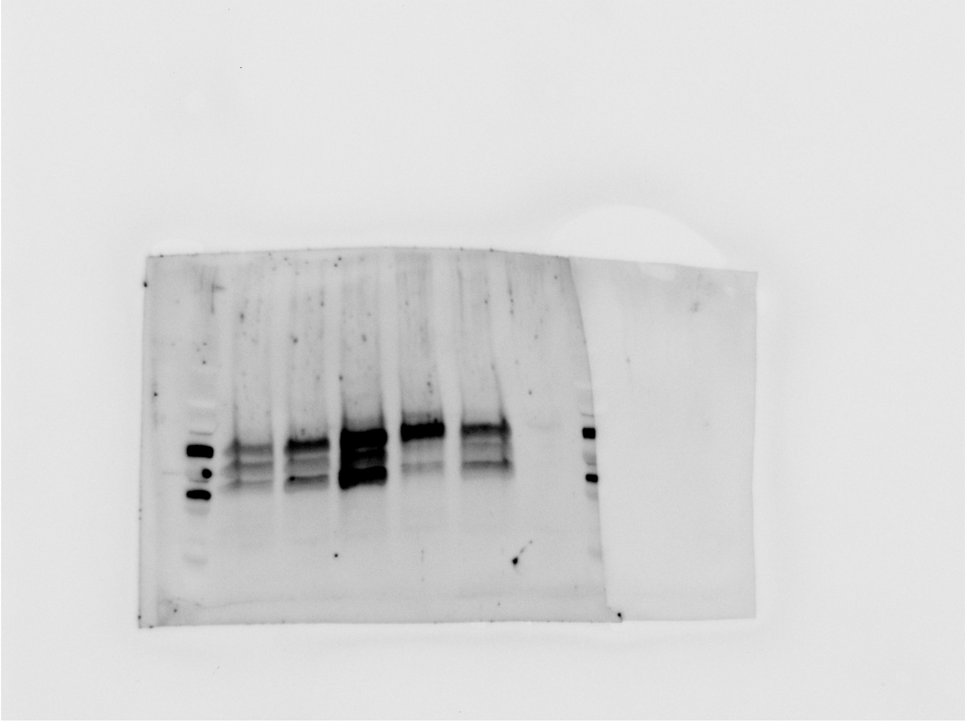

Supplement: Supplementary file 9 — Source Data [file 41467_2022_31228_MOESM9_ESM.zip › Immunoblots_NatComms.pdf]
